# Towards Iron(II) Complexes with Octahedral Geometry: Synthesis, Structure and Photophysical Properties

**DOI:** 10.3390/molecules25245991

**Published:** 2020-12-17

**Authors:** Mohamed Darari, Antonio Francés-Monerris, Bogdan Marekha, Abdelatif Doudouh, Emmanuel Wenger, Antonio Monari, Stefan Haacke, Philippe C. Gros

**Affiliations:** 1 Université de Lorraine, CNRS, L2CM, F-54000 Nancy, France; mohamed.darari@univ-lorraine.fr; 2 Université de Lorraine, CNRS, LPCT, F-54000 Nancy, France; antonio.frances@univ-lorraine.fr (A.F.-M.); Antonio.Monari@univ-lorraine.fr (A.M.); 3 Departament de Química Física, Universitat de València, 46100 Burjassot, Spain; 4 Max-Planck-Institute for Medical Research, 69120 Heidelberg, Germany; bogdan.a.marekha@gmail.com; 5 Université de Lorraine, CNRS, CRM2, F-54000 Nancy, France; abdelatif.doudouh@univ-lorraine.fr (A.D.); emmanuel.wenger@univ-lorraine.fr (E.W.); 6 Université de Strasbourg, CNRS, IPCMS, F-67034 Strasbourg, France; stefan.haacke@ipcms.unistra.fr

**Keywords:** iron (II) complexes, octahedral geometry, excited states dynamics, density functional theory, time-resolved spectroscopy

## Abstract

The control of ligand-field splitting in iron (II) complexes is critical to slow down the metal-to-ligand charge transfer (MLCT)-excited states deactivation pathways. The gap between the metal-centered states is maximal when the coordination sphere of the complex approaches an ideal octahedral geometry. Two new iron(II) complexes (**C1** and **C2**), prepared from pyridylNHC and pyridylquinoline type ligands, respectively, have a near-perfect octahedral coordination of the metal. The photophysics of the complexes have been further investigated by means of ultrafast spectroscopy and TD-DFT modeling. For **C1**, it is shown that—despite the geometrical improvement—the excited state deactivation is faster than for the parent pseudo-octahedral **C0** complex. This unexpected result is due to the increased ligand flexibility in **C1** that lowers the energetic barrier for the relaxation of ^3^MLCT into the ^3^MC state. For **C2**, the effect of the increased ligand field is not strong enough to close the prominent deactivation channel into the metal-centered quintet state, as for other Fe-polypyridine complexes.

## 1. Introduction

Ligand-field splitting is an important parameter with a deep impact on the photophysics of transition metal-based complexes. Indeed, its fine tuning is crucial to populate long-lived metal-to-ligand charge transfer (MLCT) states that are required for triggering light induced functions such as photochemically induced electron transfer [1,2,3,4,5]. The control of the ligand-field splitting is particularly challenging in iron (II) polyimine complexes where the MLCT states, first populated upon photo excitation, undergo an ultrafast deactivation into the low-lying metal-centered (MC) states, thus leading to the loss of the suitable photophysical properties [6,7].

*N*-heterocyclic carbenes (NHC) ligands, ideally combining a strong σ-donating and a weak-to-moderate π-accepting character, have been reported to destabilize the MC states over the MLCT manifold, thus impressively slowing down the deactivation pathways. In this context, tridentate pyridyldicarbene ligands with appropriate modifications of both the NHC side and the central azine have been reported by us and other groups as leading to remarkable ^3^MLCT lifetimes in the 9–32 ps range for the corresponding iron (II) complexes [4,8,9,10,11,12,13,14]. Other ligands giving rise to more rigid complexes [15] or different coordination architectures [16] have achieved impressive MLCT lifetimes beyond hundreds of picoseconds. A comprehensive review of the rapid advances in this area was recently published [17].

The ligand-field splitting can also be enhanced by reducing the angular strain around the metal center [18,19,20,21,22], i.e., when the coordination geometry is as close as possible to a perfect octahedron, as seen for example in some heavy metal complexes. For example, the angularly strained [Ru(tpy)_2_]^2+^ complex (tpy = 2,2′:6′,2′’-terpyridine) experiences an ultrafast population of MC states [23], while the weakly distorted bidentate homologue [Ru(bpy)_3_]^2+^ (bpy = 2,2′-bipyridine) [24] is strongly luminescent at room temperature with excited-state lifetimes up to 1 ms since the access to deactivating MC states is hampered [25].

In the case of Ru-based compounds, the angular strain has been minimized by switching from tridentate to bidentate ligands. We have recently shown that the use of a bidentate pyridylNHC ligands also improved notably the excited state lifetimes of iron complexes compared to their tridentate analogues [11].

However, in contrast to tridentate ligands, the dissymmetrical bidentate ligands generally form mixtures of *fac* and *mer* isomers, which can be problematic when a vectorial electron transfer is required [26]. In addition, it has also been shown that the *fac* and *mer* isomerism may lead to unexpected photophysical consequences [11,13,14]. Thus, the possibility of achieving tridentate iron complexes with reduced angular strain still appears extremely attractive for the design of photoelectrochemical active agents. While some elegant approaches to increase the ligand field using tridentate ligands have been developed [18,27,28,29] there is a lack of such advances for iron (II) complexes, and the benefit of an improved octahedral coordination on the excited state relaxation dynamics has only be explored for an Fe(II) complex with 2,6-bis(2-carboxypyridyl)pyridine ligands [30]. Here, it was shown that the higher ligand field splitting destabilizes the ^5^MC, leading to a faster deactivation of this quintet state.

**Figure 1:**
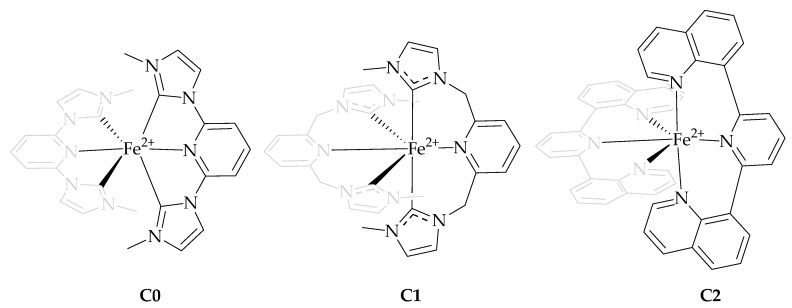
Complexes studied in this work. In all cases, counter ion is PF_6_^−^.

Herein we report the synthesis, structural characterization and photophysics of the novel homoleptic complexes **C1** and **C2** (Figure 1) based on relevant tridentate ligands selected for their ability to create an almost perfect octahedral geometry of the coordination sphere. In particular, we focus on the dqp (2,6-diquinolylpyridine) ligand, which has been reported to give a close to perfect octahedral complexes having a great impact on the MLCT lifetimes that reached the microsecond scale for the homoleptic Ru(dqp)_2_^2+^ complex (**C2**) [18]. A second ligand structure achieved by introducing methylene spacers on the 2,6-Bis(1-methylimidazolylidene) pyridine ligand has been used to make **C1**. It shall be noted that the 2,6-Bis(1-methylimidazolylidene) pyridine has been previously reported to give **C0**, a complex with a distorted octahedral Fe^2+^ coordination exhibiting MLCT lifetimes of 9 ps [8].

By such modifications, we investigated the effects induced by the optimal coordination geometry on the photophysical properties of these new iron complexes aiming to extend the excited-state lifetimes analogously to the reported case of ruthenium.

## 2. Results

### 2.1. Synthesis and Characterization

#### 2.1.1. Synthesis of Ligands and Complexes

**Scheme 1:**
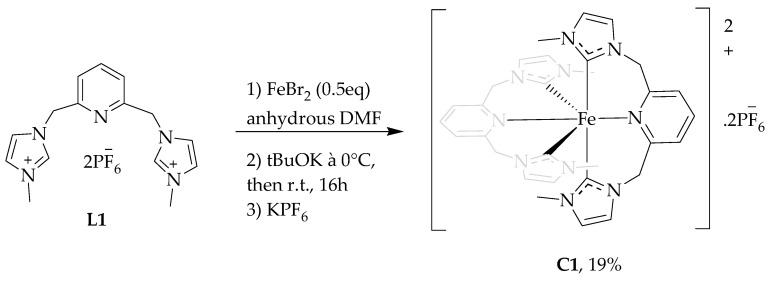
Synthesis of complex **C1**.

The access to **C1** required the synthesis of the pyridylimidazolium salt precursor **L1**. It was obtained in 80% yield from 2,6-bis(bromomethyl)pyridine and 1-methylimidazole according to the literature [31]. **L1** was subsequently involved in the coordination process by a first reaction with FeBr_2_ followed by the addition of *t*BuOK to generate the carbene (Scheme 1). When performed at room temperature, the reaction led to degradation of **L1**. However, the coordination was successfully made by decreasing the temperature to 0 °C affording **C1** in 19% yield. The low coordination yield obtained is in agreement with those obtained previously with Fe(II)NHC ligands [4,9,13].

**Scheme 2:**
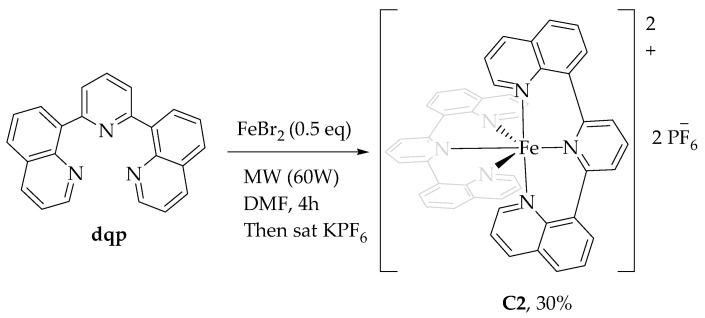
Synthesis of complex **C2**.

The diquinolinylpyridine (dqp) ligand required for the synthesis of **C2** has been obtained in 65% yield employing a Suzuki cross-coupling between 8-quinoline boronic acid and 2,6-dibromopyridine according to a reported procedure [18]. The coordination of this ligand with iron was first attempted by mixing FeBr_2_ and dqp, where regardless of the temperature and solvent used, no complex formation was detected. Switching to a microwave irradiation (60 W) in refluxing DMF for 4 h gave the expected complex **C2** in 30% yield (Scheme 2). Another iron precursor has been tested by using ammonium iron(II) sulfate hexahydrate [Fe(NH_4_)_2_(SO_4_)_2_]_6_ H_2_O. Overnight reaction at room temperature in acetonitrile gave **C2** also in 30% yield.

#### 2.1.2. X-Ray Structures

**Figure 2:**
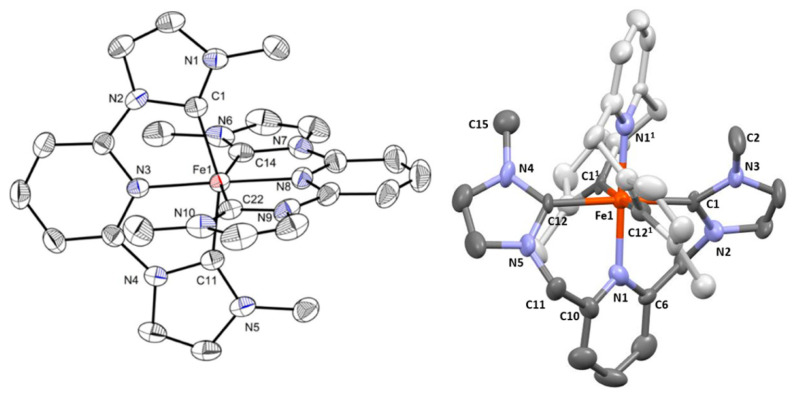
Crystallographic structures of **C0** (**left**) [8] and **C1** (**right**). Hydrogen bonds and PF_6_ molecules are omitted for clarity. Thermal ellipsoids are set to 50% probability level.

Single crystals of the two complexes suitable for X-ray diffraction analysis were obtained from slow evaporation of acetonitrile solution. X-ray crystal structures of the complexes C1 and C2 are shown in Figure 1 and Figure 2, and selected bond lengths and bond angles are given in Table 1 and Table 2.

In contrast to its parent complex C0, which has a bite angle of 158° [8], the geometry around the central iron atom in C1 is very close to fully octahedral as shown by the increased bite angles (176.1°) afforded by the flexibility provided by the methylene extension. The Fe–Pyridine bonds are longer than those of C0 (2.035 vs. 1.930 Å), which agrees with the widening of the coordination sphere in C1.

**Figure 3:**
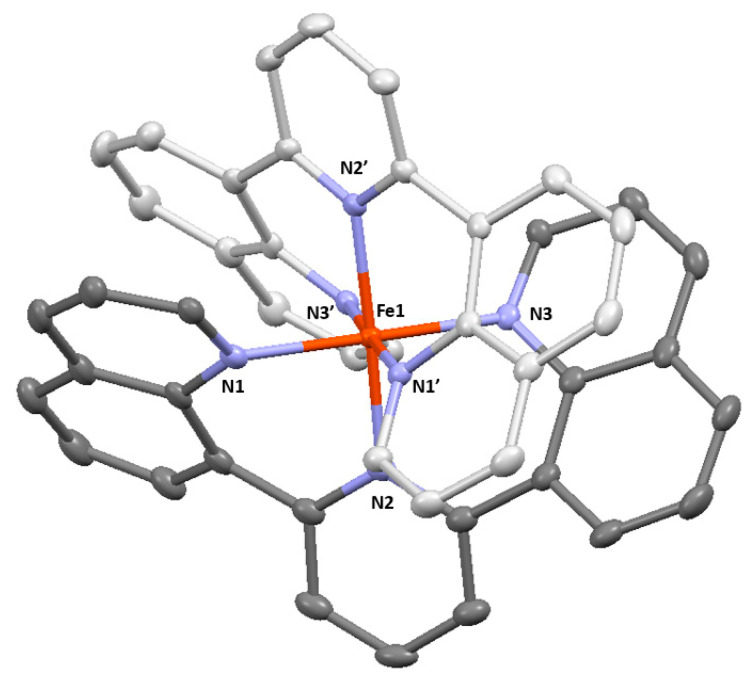
Crystallographic structure of **C2**. Hydrogen bonds and PF_6_ molecules are omitted for clarity. Thermal ellipsoids are set to 50% probability level.

Like its ruthenium homologue [18], the crystallographic angles confirm the optimization of the octahedral symmetric coordination sphere of C2 (Figure 3). Indeed, ideal coordination angles close to 90° on average have been obtained (Table 2), while maintaining bite angles close to 180°. The Fe–N (quinoline) bonds are slightly longer than the two Fe–N (pyridine) bonds due to the better charge delocalization in quinoline, increasing the electronic density and the amount of antibonding interactions with the iron d orbitals.

#### 2.1.3. Electronic and Electrochemical Properties

**Figure 4:**
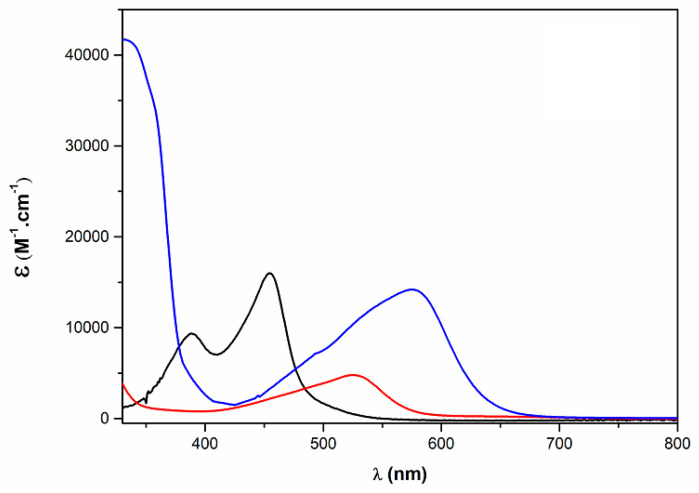
UV-Vis spectra of **C0** (**black**), **C1** (**red**) and **C2** (**blue**) in acetonitrile.

The electronic and optical properties of both complexes were investigated by means of steady-state UV-vis spectroscopy (Figure 4) and cyclic voltammetry (Table 3).

The UV-Vis spectra of both complexes present two types of absorption bands. Very intense bands are in the UV range (<350 nm) and are ascribed to bright π−π* transitions localized on the ligands. For each complex, one distinct, broader, and less intense band is found at longer wavelengths in the visible range. The absorption spectrum of **C2** presents a better spectral coverage, its absorption band at 575 nm (14,200 M^−1^ cm^−1^) is notably red-shifted by 50 nm as compared to the maximum absorption band of **C1**, which peaks at 525 nm (4800 M^−1^ cm^−1^). This bathochromic shift can be attributed to the combination of the geometric stabilization and the electron-withdrawing effects of quinolines stabilizing the MLCT states. One can note that for **C1,** the characteristic absorption band of the Fe-carbene transitions is no longer visible or resolved as it is the case in its parent complex **C0,** there with a maximum at 393 nm. This transition is most probably hidden in the broad MLCT absorption band in the 420–500 nm range. The decrease in the absorption capacity observed in **C1** could be related to the small barrier between singlet and triplet MLCT states (see Figure 8) producing an increased triplet character.

The redox properties of both complexes were measured by cyclic voltammetry with SCE as standard electrode and compared in Table 3.

For **C2**, as compared to **C0**, electrochemical measurements show an increase of the Fe^II^/Fe^III^ oxidation potential (0.96 V vs. 0.71 V) due to the π-acceptor effect of quinolines. The reduction potential is also increased (−1.35 V vs. −2.00 V), which could be ascribed in a first approximation to a lower LUMO energy level due to a greater stabilization through extended π-conjugation in the bqp ligand compared to the NHC ligand in **C0**. The lower electrochemical gap is in good agreement with the bathochromic effect observed in the UV-Vis spectra.

For **C1,** a strong decrease of the Fe^II^/Fe^III^ oxidation potential is observed compared to its parent complex **C0** (0.31 V vs. 0.71 V) which is indicative of the marked increase of the σ-donating character of the carbene moieties which are no more conjugated with the pyridine as in **C0**. However, **C1** exhibits an irreversible monoelectronic transfer at −2.05 V occurring at about the same potential as **C0** indicating for these two complexes higher energy levels of the π* orbitals than for **C2**.

### 2.2. Excited State Relaxation Dynamics

The effects of the ligand geometry of the coordination sphere on the excited state lifetimes was investigated for compounds **C1** and **C2** by femtosecond transient absorption spectroscopy (fs-TAS) and compared to **C0**, as reference [8].

#### 2.2.1. Fs-TAS of C1

**Figure 5:**
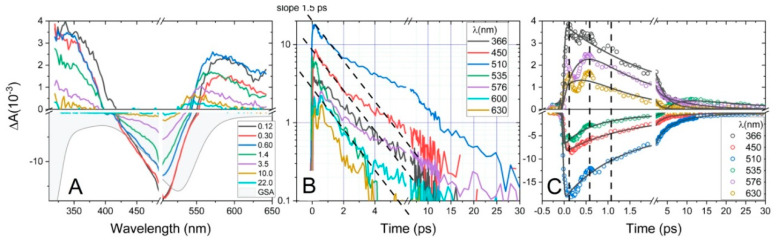
Femtosecond TAS data of **C1** in acetonitrile (ACN) after excitation at 495 nm. (**A**): Time-dependent spectra for delays in ps, as indicated in the legend. Ground state absorption (GSA) is the sign-inverted ground state absorption spectrum. Note the different scales for the positive and negative parts of ΔA. The 480–505 nm range is disregarded due to excessive pump light scattering. (**B**): Decay transients at selected wavelengths (cf. legend) plotted on a semi-log scale with a break in the time axis at 6 ps. GSB traces are sign inverted. Dashed lines are guides-to-the-eye with a 1.5 ps decay slope, highlighting that the initial fast decay of the signals is in that time range. When the signal-to-noise is large enough, an additional 10–15 ps decay emerges. (**C**): Decay transients at selected wavelengths (cf. legend) plotted on a linear scale with a break in the time axis at 2.0 ps. Dashed vertical lines mark the occurrence of oscillatory maxima in the excited state absorption (ESA) with a period of ≈0.5 ps. Solid black lines are the best fits to the data, without accounting for the oscillatory part. Note the different scales for the positive and negative parts of ΔA. Transients are averaged over ±5 nm.

**C1** dissolved in acetonitrile (ACN) was excited with ≈50 fs pump pulses at 495 nm, and the pump-induced absorbance changes ΔA, with no more than 10% ground state depletion, were probed by a white-light continuum generated in a 2-mm thin CaF_2_ plate in the range of 320–640 nm. Figure 5 displays the time-dependent ΔA, and the sign-inverted ground state absorption spectrum (GSA) for comparison. The TAS data show at all times a negative ground state bleach (GSB) in the range of 410–540 nm at early delays, with a peak at 507 nm. Positive ΔA due to excited state absorption (ESA) is identified in the blue part of the GSB, below 400 nm, and in the red, above 520–540 nm. Note that both ESA bands have significant overlap with the GSB, since the latter is visible only in a limited range of the GSA spectrum, due to the larger absorption cross section of ESA in the ranges where ΔA > 0. Clear spectral evolution occurs on a sub-300 fs time scale in the blue ESA band, shifting the ESA-GSB crossover around 400 nm by 20 nm, and in the red ESA during a longer time period, i.e., the first 10 ps (25 nm red-shift). This indicates that different species contribute to the ESA, populated along a relaxation scheme we will identify in what follows.

Another interesting observation is related to periodic modulations in the ESA amplitude. Figure 5C highlights these for the blue and red ESA (366, 576, 630 nm), and for the longer wavelength part of the GSB (510 and 535 nm), but not for 450 nm. The oscillation period is difficult to determine precisely as damping is strong, but it can be determined to be of the order of 0.5 ps. Maxima of the oscillatory features in ESA correspond to minima at the wavelengths of negative ΔA. Keeping in mind that the latter is a result of overlapping ESA and GSB, one can conclude, in particular for λ > 450 nm, that the modulations are in the ESA signal only. In other words, the excited state transition dipole moment is modulated by a strongly damped but coherent vibrational motion with a frequency of ≈60 cm^−1^. See SI for a more quantitative analysis, and higher frequency ground state oscillations.

Regarding the excited state lifetimes and relaxation scenario, Figure 5B plots kinetic traces on a semi-log scale, so as to highlight the components of exponential decay. Dashed black lines with a slope of 1.5 ps overlay with the initial portion of most of the kinetic traces. When the signal-to-noise ratio is high enough, an additional slower component, 10–15 ps, is observed (450, 510, 576 and 600 nm). All kinetic traces can be well fitted by a sum of three exponentials with wavelength-dependent amplitudes and decay constants, $\sum_{i=1}^{3}A_{i}\left(\lambda \right)e^{-t/\tau_{i}\left(\lambda \right)}$, convoluted with a Gaussian instrument response function (width 50 fs). The results of single wavelength fits are displayed as solid black lines in Figure 5C. They reveal a rise time, in the ≈0.3 ps range, for the red ESA part (576 and 630 nm). Table S16 in the Supplementary Materials gives the fit parameters for individual wavelengths. The fastest ESA decay and GSB recovery time is τ_2_ = 0.8–1.5 ps, and the second decay is found by τ_3_ = 7–17 ps, with an overall 8–10 times smaller amplitude than τ_2_.

**Figure 6:**
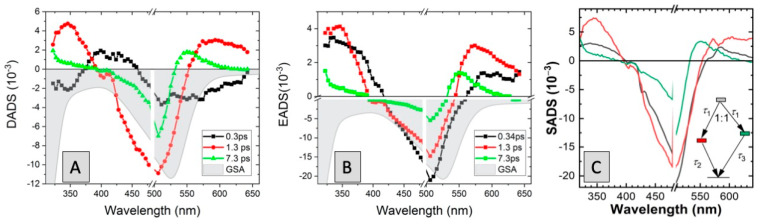
Global and target analysis of the TAS data of compound **C1** in ACN. Lifetimes as indicated in the legend, see text for interpretation. (**A**): Decay-associated difference spectra (DADS) identifying the three global lifetimes 0.3, 1.3 and 7.3 ps, with 10% error bar. (**B**): Evolution-associated difference spectra (EADS) for a fully sequential model, Scheme 2. (**C**): A parallel relaxation model, Scheme 1, with a 50/50 partition between the excited state triplets T_1_ and T_2_.

Global analysis, i.e., fits of the full data set, but with wavelength-independent lifetimes, $\sum_{i=1}^{3}A_{i}\left(\lambda \right)e^{-t/\tau_{i}}$, were carried out, using OPTIMUS [32] and the results displayed as decay-associated difference spectra (DADS,$\;A_{i}\left(\lambda \right)$) in Figure 6A. The oscillatory components are neglected in these fits, but they represent the qualitative trends of Figure 5. The validity of such global fitting is assessed in detail in the Supplementary Materials (cf. Figure S7). First, the negative amplitude of the 0.3-ps DADS in the blue and red ESA regions reflect the observed rise. Likewise, its positive amplitude in the 380–450 nm range is consistent with the slightly rising GSB signal in this time and wavelength ranges (Figure 5C). Second, the double exponential decay character of both ESA and GSB is reliably reproduced in the DADS of τ_2_ = 1.3 ps and τ_2_ = 7.3 ps. We find that the amplitude of τ_2_ is approx. 10 times larger than the one of τ_3_ in the blue ESA band and above 580 nm. However, since the ESA amplitudes depend on the respective cross sections and overlap with the GSA, it is not straightforward to conclude about the relative populations of these channels.

What is the excited state relaxation scenario emerging from these spectroscopic data? The sub-picosecond τ_1_ is usually assigned to the transition from the optically excited ^1^MLCT to ^3^MLCT and vibrational relaxation of the latter, reflected in the ultrafast rise of ESA below 380 and above 500 nm. The positive DADS of τ_1_ in the 380–450 nm range can be related to a reshaping of ESA, i.e., vibrational relaxation of ^3^MLCT. On the other hand, the DADS of τ_2_ and τ_3_ are very similar to the situation we recently reported for complexes with bidentate ligands [14], where two excited states seem to co-exist, and is rather at odds with the single excited state scenario we reported for tridentate Fe–NHC complexes [12]. Indeed, for the latter τ_3_ was dominant in intensity and τ_2_ with minor amplitude rather related to structural relaxation in the single excited state. Hence, the TAS data are consistent with the existence of two excited triplet states, T_1_ and T_2_, populated via relaxation from ^1^MLCT and subsequent vibrational cooling during τ_1_. Their precise nature in terms of diabatic states (MLCT, MC or mixtures of both) cannot be determined from the TAS difference spectra, and will be addressed by TD-DFT calculations (Section 2.3). However, the experimental data suggest a parallel decay back to the ground state S_0_, according to the following Equation (1):

(1) $${}^{1}{MLCT}^{+}\overset{<IRF}{\to }\{\begin{array}{c} {}^{3}{{MLCT}_{1}}^{+}\\ {}^{3}{{MLCT}_{2}}^{+} \end{array}\overset{\tau_{1}}{\to }\{\begin{array}{c} T_{1}\overset{\tau_{3}}{\to }S_{0}\\ T_{2}\overset{\tau_{2}}{\to }S_{0} \end{array}$$

Here, the “+” index denotes vibrationally excited electronic states, inevitably present in the first hundreds of femtoseconds after laser excitation. This scenario was tested with a 50/50 population of T1 and T2 and the species-associated difference spectra (SADS) obtained using GLOTARAN [33] are given in Figure 6C. They provide reasonable difference spectra for these triplet states, with clearly separated bands and transition energies. In the absence of any further knowledge, e.g., from simulations about the ESA spectra of these states, Equation (1) appears to be a viable interpretation of the data. Note that similar excited state branching scenarios, though with different lifetimes and state assignments, were recently published for Fe–NHC complexes with four carbene ligands [34,35].

On the other hand, an alternative purely sequential relaxation scheme can be postulated according to Equation (2):

(2) $${}^{1}{MLCT}^{+}\overset{<IRF}{\to }{}^{3}{MLCT}^{+}\overset{{\;\tau }_{1}\;}{\to }T_{1}\overset{{\;\tau }_{2}\;}{\to }X\overset{\;\tau_{3\;}}{\to }S_{0}$$

The corresponding evolution-associated difference spectra (EADS) are given in Figure 6B. The excited state relaxation populates a triplet state T_1_ within 0.3 ps, which decays on a 1.3 ps timescale into a state X, which has a 7.3 ps lifetime. While it cannot be excluded that X is an excited state of unknown nature, the EADS of this species suggests its absorption spectrum to be very similar to the GSA of **C1**, the only differences being a slight 20–30 nm red-shift of the main transitions (see Supplementary Materials). It is therefore very likely that, in this sequential scenario, X represents a vibrationally excited, “hot” form of S_0_. Further information comes from TD-DFT calculations, which will be discussed in Section 2.3.

#### 2.2.2. Fs-TAS of **C2**

**Figure 7:**
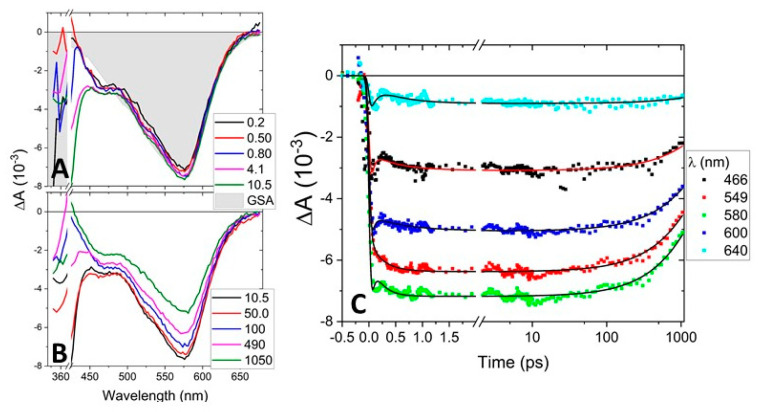
Transient absorption data of **C2**. (**A**,**B**) time-resolved ΔA spectra at delays indicated in the legend (in ps). The region 365–425 nm is omitted due to excessive pump laser scattering. (**C**): Kinetic traces at different wavelengths in the main bleach band, together with bi-exponential fits (see text for details). The sharp features at ≈0.1 ps are most likely due to cross phase modulation (“coherent artefact”).

**C2** dissolved in ACN was excited with ≈50 fs pump pulses at 400 nm, and the pump-induced absorbance changes ΔA, in the range of 350–650 nm are displayed in Figure 7A,B together with the sign-inverted ground state absorption spectrum (GSA) in panel A. The TAS data show a negative ground state bleach (GSB) at all delays probed and over the full wavelength range. A small positive ΔA on the 0.2 ps spectrum may be due to excited state absorption (ESA) from an early decaying state, but since these data are not corrected for cross-phase modulation around time zero, the assignment is unclear. As shown by the kinetic traces (Figure 7C), the bleach signal increases slightly between time zero and ≈1.0 ps, and decays thereafter on a time scale longer than the observation window (1.1 ns). Double exponential fits of traces at individual wavelengths, displayed in Figure 7C, yield a GSB rise time τ_1_ of 0.2–0.5 ps, and a decay time τ_2_ = 3.2 ± 0.5 ns.

Cast in a global lifetime analysis, values of τ_1_ = 0.45 ± 0.1 ps, and τ_2_ = 3.0 ± 0.1 ns are found. The DADS and the SADS using a sequential relaxation scheme are displayed in Figure S9. The lifetime and ΔA spectrum of the long-lived species is consistent with the one observed for the ^5^MC quintet state of [Fe(tpy)_2_]^2+^ in H_2_O [36], in particular the very weak ESA, if any, in the red part of the spectrum distinguishes it from an ^3^MLCT state (cf. **C1**, and other Fe–NHC complexes). Note that the negative ΔA signal level <360 nm is much smaller than the expected GSB (inverted GSA), pointing to absorption of the MC quintet, in good agreement with reports probing ^5^MC of [Fe(tpy)_2_]^2+^down to 290 nm [37]. In analogy to other polypyridyl complexes of Fe(II), the data are consistent with an ultrafast spin crossover transition from the ^1/3^MLCT manifold, identified by the SADS of τ_1_ (Figure S3). Hence, the relaxation scheme for **C2** is:

(3) $${}^{1}{MLCT}^{+}\overset{<IRF}{\to }{}^{3}{MLCT}^{+}\overset{{\;\tau }_{1}=0.45\;ps\;}{\to }{}^{5}MC\overset{\;\tau_{2}\;=\;3.0ns}{\to }S_{0}$$

It must be noted, however, that the ultrafast relaxation into ^5^MC leads necessarily to a vibrationally excited quintet. The vibrational relaxation time on a similar spin cross-over complex was found to be in the 10 ps range, as identified by fs mid-IR spectroscopy [38]. Apparently, these cooling processes go unnoticed or do not show up with significant spectral changes in the near UV/VIS for **C2.**

### 2.3. Excited-State Decay Mechanism Based on TD-DFT Calculations

**Figure 8:**
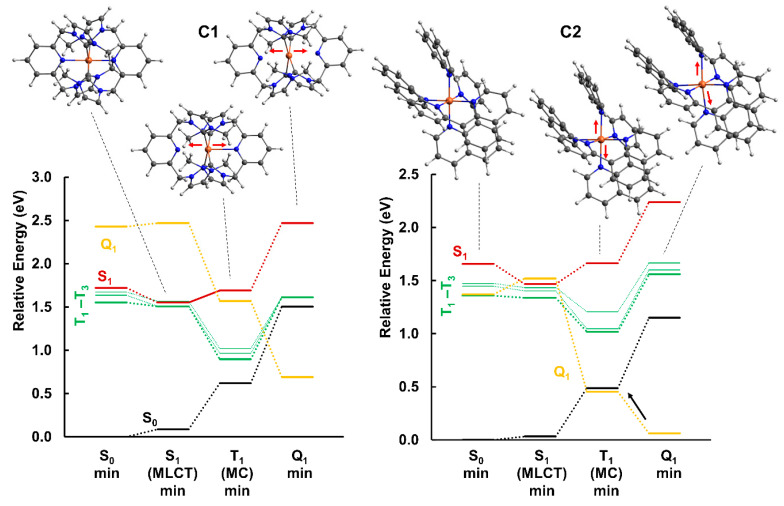
Relative energies of the ground state (S_0_) and the lowest-lying singlet (S_1_), triplet (T_1_) and quintet (Q_1_) excited states of complexes **C1** (**left**) and **C2** (**right**). T_2_ and T_3_ states are also shown in thinner green lines. The dashed lines represent non-connected paths between the different geometries. Red arrows in the 3D molecular representations highlight the Fe–N bonds with the greatest elongations during the excited-state decay (Fe–N1 and Fe–N1′ for **C1**, Fe–N1 and Fe–N3 for **C2**, the values are summarized in Table 4). All energies are relative to the ground state at the corresponding S_0_ min structure.

The most relevant electronic states involved in the photoresponses of **C1** and **C2** are displayed in Figure 8. We have adopted the computational methodology used in our previous works on Fe–NHC complexes with bidentate [11,13,14] and tridentate ligands [12] in order to obtain fully comparable results.

As in other transition metal complexes, light absorption populates bright high-lying ^1^MLCT states of **C1**. As commented in the previous sections, ultrafast intersystem crossing leads to the population of the triplet ^3^MLCT manifold in a sub-picosecond regime, followed by vibrational relaxation to the lowest-lying ^3^MLCT state (T_1_). From a potential energy surface (PES) perspective, all these phenomena take place in regions close to Franck-Condon and S_1_ min areas, in which the ^1^MLCT and ^3^MLCT states are also close in energy (see Figure 8). The term S_1_ min areas refer to the PES regions where the energy of the S_1_ state is minimum. On longer time scales, the triplet state must evolve to reach non-adiabatic crossing points with the ground state that mediate non-radiative decay channels. These mechanisms are intrinsically different between Fe–NHC complexes with bidentate [11,13,14] and tridentate [12] ligands and have different implications for the longer ESA components τ_2_ and τ_3_. In the former type of complexes, relaxation of the lowest-lying triplet state T_1_ leads to a ^3^MC region through a barrierless path encompassing an extended area of spin-crossover character, i.e., an area where S_0_ lies above T_1_, in which the system can be trapped for several picoseconds [13]. On the contrary, in complexes with tridentate ligands, the T_1_ relaxation leads to a well-defined ^3^MLCT minimum close to the Franck-Condon region [12]. The stretching of Fe–N bonds, always involved in the stabilization of the dissociative ^3^MC states, is clearly the dominant degree of freedom in driving the excited state decay. Due to the different topology of the PESs, Fe–N bonds are spontaneously stretched in the triplet manifold of complexes with bidentate ligands, while this molecular motion has an energy cost in Fe–NHC systems with tridentate ligands, explaining the triplet lifetimes of tens of picoseconds in the latter [12].

**Figure 9:**
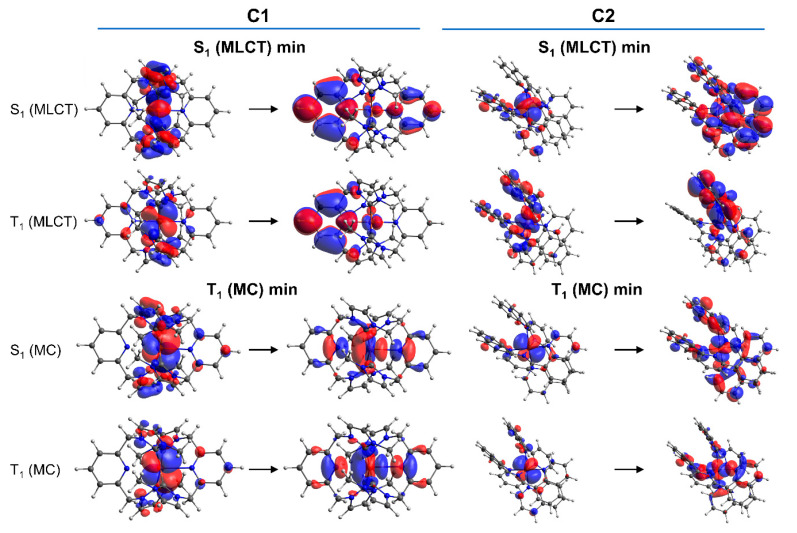
Natural transition orbitals (NTOs) of the S_1_ and T_1_ states at their corresponding optimized geometries for **C1** (**left**) and **C2** (**right**).

Despite the tridentate coordination, the ES decay mechanism of **C1** lies in between these two extreme cases and somehow closer to the bidentate type mechanism. The T_1_ min has a clear ^3^MC nature (see Figure 9), with Fe–N distance stretched up to 2.60 Å (see Table 4). As a matter of fact, the T_1_ min geometry is close to but does not perfectly represent a singlet-triplet crossing (STC) with S_0_, since the singlet-triplet energy gap is still 0.28 eV, although this relatively low energy splitting suggests that the STC must be in the surroundings. As a matter of fact, the T_1_ potential energy surface shown in Figure S10 confirms the access to the singlet-triplet crossing region with the ground state with an energy barrier < 0.05 eV. On the other hand, the energy difference between the T_1_–T_3_ states at the T_1_ min region is smaller as compared to that of previous bidentate Fe–NHC complexes, supporting a possible sequential decay mechanism (T_1_/T_2_/T_3_→S_0_) in which τ_2_ is ascribed to the population of the ground state and τ_3_ belongs to vibrational relaxation of S_0_. A parallel mechanism like the one proposed for pure bidentate complexes (see Scheme 1 and refs [11,13,14,39], in which the T_1_ and T_2_ states are associated to different ES paths operating in parallel, cannot be excluded, although it seems less plausible in this particular case given the T_1_–T_3_ energy degeneracy at S_1_ min and T_1_ min regions.

The analysis of the photodynamics of **C1** is also of particular value since it reinforces important general conclusions about the photophysics of this type of Fe(II) complexes. In particular, the flexibility added by the presence of alkyl spacers between the pyridine and imidazole rings opens ES decay paths that are forbidden for the more rigid tridentate complex, that is, longer Fe–N elongations leading to accessible ^3^MC minima. This is reflected in the TAS signals (two long components in bidentates vs. one component in tridentates) and in the photochemical landscape shown in Figure 8. Hence, a subtle equilibrium between the ideality of the octahedral coordination, destabilizing the ^3^MC states in the Franck-Condon region, and limited flexibility, hampering an efficient population of ^3^MC states at regions far from the Franck-Condon area via Fe–N elongations, should be carefully considered when designing photoactive iron complexes.

On the contrary, the ES decay mechanism of **C2**, as also highlighted by TAS results, follows a completely different scenario. A major difference as compared to **C1** is the pivotal role played by the much more stable Q_1_ state, which is degenerated with T_1_ already at the Franck-Condon geometry (see Figure 8). Whereas the optimization of the S_1_ (^1^MLCT) state destabilizes Q_1_, this state is on the contrary greatly stabilized with the Fe–N stretching associated to the T_1_ relaxation. Indeed, the Q_1_ state becomes degenerated with the ground state at the T_1_ min region, which is of ^3^MC nature (see Figure 9). Full optimization of the Q_1_ state gives the Q_1_ min, in which the state is at a relative energy close to that of the ground state at its corresponding equilibrium geometry, hence fully justifying the observed light-induced spin crossover capabilities of **C2**.

Therefore, as stated above, the data presented in Figure 8 indicate that the Q_1_ state is the main actor of **C2′**s photoresponse, in coherence with the TAS analysis depicted above. This state is populated in the Franck-Condon region or in the surroundings in ~0.3 ps and is expected to rapidly evolve towards the Q_1_ min region. At this region, the S_0_ is not accessible (note that its relative energy is of 1.15 eV, more than 1 eV above Q_1_), and therefore one can expect a significant barrier (at least 0.5 eV, according to the solid black arrow shown Figure 8) to repopulate the ground state. This description is in good agreement with the long 3.0 ns lifetime measured experimentally for the quintuplet state.

## 3. Materials and Methods

### 3.1. General Information

Solvents and commercially available reagents were used as received. Thin layer chromatography (TLC) was performed by using silica gel 60 F-254 (Merck) plates and visualized under UV light. Chromatographic purification was performed by using silica gel 60 (0.063–0.2 mm/70–230 mesh). ^1^H (400 MHz) and ^13^C NMR (100 MHz) spectra were taken on a DRX400 Bruker spectrometer at ambient temperature. The chemical shifts (δ) were calibrated by using either tetramethylsilane (TMS) or signals from the residual protons of the deuterated solvents and are reported in parts per million (ppm) from low to high field. High-resolution mass spectrometry (HRMS) data was obtained by using Bruker micrOTOF-Q spectrometer. UV vis spectra were recorded in a 1 cm path length quartz cell on a LAMBDA 1050 (PerkinElmer), spectrophotometer. Cyclic voltammetry was performed on a Radiometer PST006 potentiostat using a conventional three-electrode cell. The saturated calomel electrode (SCE) was separated from the test compartment using a bridge tube. The solutions of studied complexes (0.2 mM) were purged with argon before each measurement. The test solution was acetonitrile containing 0.1 M Bu_4_NPF_6_ as supporting electrolyte. The working electrode was a vitreous carbon rod (1 cm^2^) wire, and the counter-electrode was a 1 cm^2^ Pt disc. After the measurement, ferrocene (Fc) was added as the internal reference for calibration. All potentials were quoted versus SCE. In these conditions the redox potential of the couple Fc+/Fc was found at 0.39 V. In all the experiments the scan rate was 100 mV.s^−1^.

### 3.2. Xray Diffraction

Two single crystals of complexes **C1** and **C2** were selected and measured with a Rigaku Oxford Diffraction SuperNova 4-circles diffractometer. This diffractometer was equipped with a microfocus X-ray source with a Molybdenum anode (MoKα, λ = 0.71073 Å) and an Atlas CCD detector. An Oxford Cryosystems Cryostream nitrogen blower was used to fix the sample temperature at 100 K for C1 and 110 K for C2. Using Olex2 software [40], structures were solved with XT structure solution program [41] and structures were refined with the XL refinement package [41] by using Least Square minimization. CCDC 1992132 (C1) and CCDC 1982173 (C2) contain the supplementary crystallographic data for this paper. These data are provided free of charge by the Cambridge Crystallographic Data Centre.

### 3.3. Ultrafast Transient Absorption Spectroscopy

An amplified 5kHz Ti: sapphire laser generates 30 fs 0.5 mJ pulses to pump a commercial optical parametric amplifier (TOPAS: Light Conversion), from which 50–60 fs pulses are derived to excite the samples: 495 nm for **C1**, and 400 nm for **C2**. A white light continuum is generated in a 2 mm thick CaF_2_ crystal, mounted on an oscillating loudspeaker to reduce photo-damage. The white light is optimized for minimized fluctuations (rms < 0.4% at 220 Hz). The beam is split in two: the probe that is sent through the sample, and a reference beam is used for measuring and compensating the white-light intensity fluctuations. The polarization of the probe beam is set at magic angle (54.7°) with respect to the pump. A 1 mm path length cuvette in fused silica contains the complexes dissolved in CH_3_CN. Time-resolved spectra I (λ) are acquired in a range ≈300 nm wide window with an adjustable central wavelength as a function of pump-probe delay, by a combination of 25 cm focal length spectrometer (resolution 2 nm) and a Peltier-cooled CCD with 220 Hz acquisition rate. A second chopper blocks the pump beam at 110 Hz and a home-made software computes and displays the differential spectra $\Delta A=-log_{10}\left(\frac{I_{pump-on}}{I_{pump-off}}\right)$. A solvent-only sample is measured, and the data are processed to (i) remove a residual signal background at negative delay times, (ii) the solvent Raman signal and the coherent interactions of the pump and probe in the cell (except for C2), (iii) to correct for the group velocity dispersion of the probe beam, characterized in the solvent-only data set. The temporal resolution is determined by the 50±5 fs FWHM of the solvent Raman response. Data are fitted at individual wavelengths according to $\Delta A\left(\lambda ,t\right)=IRF\left(t_{0},\sigma \right)⊗\left(A_{0}+\sum_{i=1}^{n}A_{i}\left(\lambda \right)e^{-t/\tau_{i}\left(\lambda \right)}\right)$, (1) a sum of n-exp functions and an un-resolved contribution (A0), convoluted with a Gaussian instrument response function (IRF, σ = 35–50 fs, FWHM). Although data are chirp-corrected and t0, is adjustable for each wavelength. Global and target analysis was performed with the software packages OPTIMUS [32] and GLOTARAN [33].

### 3.4. Computational Details

The computational protocol has been widely validated in previous works and it will be only summarized here [11,12,13,14,39,42]. Unless otherwise stated, all calculations have been performed using the Gaussian 16 program [43]. The ground state geometries have been optimized using the DFT/B3LYP method, whereas the S_1_ state has been optimized using TD-DFT in combination with the pure HCTH functional [44]. The T_1_ and Q_1_ states have been optimized using the unrestricted uDFT/HCTH method. All optimizations have been performed employing the 6-31+G(d,p) basis set. Later, single-point calculations with the larger 6-311G(d,p) basis set have been performed on top of the converged geometries to obtain the final energies displayed in Figure 8. The energies of S_1_ and T_1_ have been obtained with the TD-DFT/HCTH method, while the energies of Q_1_ have been determined making use of the uDFT/HCTH ansatz. Solvent effects (acetonitrile) have been included by means of the polarizable continuum model. The Tamm-Dancoff approximation has been used in all TD-DFT calculations [45]. For **C2**, due to the inherent π-stacking interaction of the ligands, empirical dispersion has been included in all calculations through Grimme’s original D3 damping function [46]. Analysis of the nature of the excitations (Figure 9) have been performed by computing the natural transition orbitals (NTOs) [47] with the NANCY_EX code [48,49].

### 3.5. Synthesis of Ligands and Complexes

#### 3.5.1. Synthesis of Ligands

Synthesis of **L1** [31]. A solution of 2,6-bis(bromomethyl)pyridine (1.337 g, 5.0 mmol) and 1-methylimidazole (0.831 g, 10.0 mmol) was stirred in dioxane (80 mL) at 100 °C for 12 h. After cooling, the formed solid was collected and purified by repetitive precipitation from MeOH/Et2O mixtures and finally by recrystallization from CH_2_Cl_2_/Et_2_O. The product was then collected and solubilized in a small amount of water, then precipitated using a saturated aqueous solution of KPF_6_ to give **L1** (2.27 g, 80%). ^1^H-NMR (CD_3_CN, 400 MHz): δ 8.47 (s, 2H), 7.90 (t, *J* = 7.8 Hz, 1H), 7.44 (d, *J* = 7.8 Hz, 2H), 7.35 (s, 4H), 5.38 (s, 4H), 3.85 (s, 12H) ppm.

Synthesis of dqp [18]. An oven-dried flask was charged with 8- quinoline boronic acid (413 mg, 2.39 mmol), 2,6-dibromo-pyridine (265mg, 1.12mmol), Pd(dba)2 (13mg, 0.023mmol), 2-dicyclohexylphosphino-2’,6´-dimethoxybiphenyl (0.019 g, 0.046 mmol) and grinded K_3_PO_4_ (2.27 g, 10.7 mmol). The flask was evacuated and charged with N_2_. Dry toluene (10 mL) was added via a syringe and the resulting suspension was stirred at 100˚C for 15 h. The mixture could cool to room temperature, diluted with CH_2_Cl_2_ (30 mL) and filtered. The solvent was removed and the remaining solid purified by column chromatography using silica gel and 2.5% MeOH/CH_2_Cl_2_ as eluent to give dqp as an off-white solid (244mg, 65%). ^1^H-NMR (CDCl_3_, 400 MHz,): δ 9.00 (dd, *J* = 4.2, 1.9 Hz, 2H), 8.28 (dd, *J* = 7.3, 1.5 Hz, 2H), 8.24 (dd, *J* = 8.3, 1.9 Hz, 2H), 8.13 (d, *J* = 7.7 Hz, 2H), 7.96 (t, *J* = 7.7 Hz, 1H), 7.89 (dd, *J* = 8.2, 1.5 Hz, 2H), 7.67 (dd, *J* = 8.2, 7.3 Hz, 2H), 7.46 (dd, *J* = 8.3, 4.2 Hz, 2H) ppm.

#### 3.5.2. Synthesis of Complexes

Synthesis of **C1**. To a solution of **L1** (0.129 g, 0.231 mmol) in 2 mL of anhydrous DMF was added FeBr_2_ (15 mg, 0.115 mmol) and degassed the mixture with N_2_ for 10 min. Then *t*-BuOK was added (104 mg, 0.927 mmol) to the above mixture and stirred at 0 °C and temperature left slowly rising to room temperature. A saturated solution of KPF_6_ was added (10 mL), and the precipitate was collected by filtration. Then the crude was further purified on silica gel column chromatography using acetone/H_2_O/KNO_3_ (sat) = 10:3:3 mixture. The pink fraction was collected and after the evaporation of acetone, the left solution was treated with a saturated solution of KPF_6_. **C1** (20 mg, 19%) was finally obtained after filtration. ^1^H-NMR (CD_3_CN, 400 MHz): δ 7.58 (t, 2H, *J* = 7.7 Hz), 7.30 (d, 4H, *J* = 7.7 Hz), 7.07 (d, 4H, *J* = 1.9 Hz), 6.67 (d, 4H, *J* = 1.9 Hz), 5.41 (s, 4H), 3.40 (s, 4H), 2.05 (s, 12H) ppm. ^13^C-NMR (CD_3_CN, 100 MHz): δ 191.4, 157.3, 137.3, 126.3, 124.2, 120.1, 53.5, 34.5. HR-MS (ESI) calcd for C_30_H_34_FeN_10_P_2_F_12_ m/z = 295.1153 [M – 2 PF_6_]^2+^. Found: 295.1145.

Synthesis of **C2**. A 5 mL microwave tube was charged with dqp (65 mg, 0.195 mmol) and FeBr_2_ (20mg, 0.093 mmol). After addition of DMF (3 mL) the tube was sealed and heated for 4h using microwave heating (60W). A saturated solution of KPF_6_ was added (10 mL), and the precipitate was collected by filtration. Then the crude was further purified on silica gel column chromatography using Acetone/H_2_O/KNO_3_ (sat) = 10:3:3 mixture. The dark pink fraction was collected and after the evaporation of acetone, the left solution was treated with a saturated solution of KPF_6_. **C2** (28 mg, 30%) was finally obtained after filtration. ^1^H-NMR (400 MHz, CD3CN): δ 8.17 (dd, *J* = 8.2, 7.8 Hz, 2H), 8.07 (m, 8H), 7.89 (m, 4H), 7.73 (dd, *J* = 7.5, 1.3 Hz, 4H), 7.68 (dd, *J* = 8.3, 1.3 Hz, 4H), 7.45 (dd, *J* = 8.3, 7.5 Hz, 4H), 7.05 (dd, *J* = 7.9, 5.3 Hz, 4H) ppm. ^13^C-NMR (DMSO-d6, 100 MHz): δ 164.50, 164.25, 161.34, 149.95, 138.89, 138.68, 132.36, 130.84, 128.10, 127.88, 127.32, 126.25. HR-MS (ESI) calcd for C_46_H_30_FeN_6_P_2_F_12_ m/z = 361.0935 [M – 2 PF_6_]^2+^. Found: 361.0944.

## 4. Discussion and Conclusions

The present study of Fe(II) homoleptic tridentate complexes **C1** and **C2** with octahedral coordination symmetry aimed at providing evidence for the effect of a larger ligand field splitting on the excited state (ES) dynamics, in particular aiming at obtaining longer-lived ^3^MLCT states. The change in geometry had a positive effect on the absorption properties inducing notable red shifts of the Fe-pyridine MLCT absorption bands compared with the non-octahedral **C0**, in agreement with the decrease of the HOMO-LUMO gap also confirmed by cyclic voltammetry. However, as pointed out above, the ligand modifications also bring about a larger conformational flexibility, the effect of which appears to dominate the ES quantum dynamics.

**Figure 10:**
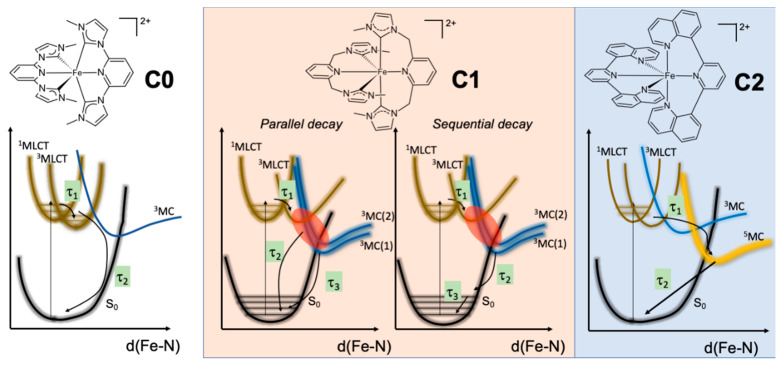
Excited state relaxation schemes for the three complexes **C0, C1** and **C2,** as inferred from TAS data and TD-DFT calculations. Lifetimes and arrows identify the relevant steps of internal conversion and intersystem crossing. (**Left**): in **C0**, the rigid tridentate structure imposes a slight barrier on the ^3^MLCT–^3^MC relaxation and stabilizes ^3^MLCT for 9–10 ps [8]. (**Center**): the larger ligand flexibility in **C1** reduces this barrier and leads to a region of adiabatic mixing between ^3^MLCT and ^3^MC (red ellipse). The sketch labeled “parallel decay” refers to Equation (1) (Section 2.2.1). However, our calculations indicate that one single triplet state minimum of ^3^MC character is transiently populated (Figure 8) hinting to the “sequential decay” scenario being more plausible (Equation (2), Section 2.2.1). (**Right**): **C2** is a photo-induced spin crossover complex like [Fe(tpy)_2_]^2+^. Note that for **C0,** the recently revealed parallel population of ^3^MLCT and ^3^MC [35] is not represented for simplicity.

When comparing **C1** with the proto-typical **C0** ([Fe(bmip)_2_]^2+^) two important differences are observed. First, the ES lifetime of **C1** is roughly six times shorter than that of **C0**. The TD-DFT calculations indicate that, due to the higher ligand flexibility, the barrier hindering the relaxation of ^3^MLCT into the metal-centered ^3^MC in [Fe(bmip)_2_]^2+^ is lowered in **C1**, leading to a faster ES deactivation (Figure 10 center). The TD-DFT calculations indicate that, most likely, one single triplet state minimum of ^3^MC character is transiently populated (Figure 8) hinting to the “sequential decay” scenario being more plausible. Secondly, a strongly damped excited state vibrational coherence is observed with a frequency of 55–65 cm^−1^, in addition to ground state vibrations in the range of ≈350 cm^−1^ (cf. Figure S8). The higher frequency is in the usual range of Fe–N or Fe–C stretch vibrations, and the 60 cm^−1^ is similar to the low frequency breathing mode identified for **C0** at 110 cm^−1^ [50]. It was recently identified to be associated with vibrationally coherent population of ^3^MC through ultrafast branching from the ^1^MLCT manifold in **C0 [36]**. We rather attribute it to the initially populated ^3^MLCT. In any case, its existence and lower frequency point to a higher flexibility of the ligands of **C1** as compared to **C0**. For **C2**, the relaxation from ^3^MLCT into the metal-centered ^3^MC and ^5^MC states (Figure 10, left) seems to be almost unaffected since the 0.45 ps lifetime is only slightly longer than the one observed for [Fe(tpy)_2_]^2+^ [8].

In summary, the comparison of the photophysics of **C1** and **C2** evidences important aspects about the Fe(II) coordination sphere directly impacting ES decay mechanisms and ES lifetimes of Fe–NHC complexes. The main conclusion is that the destabilization of ^3^MC states at the Franck-Condon region does not guarantee longer triplet MLCT lifetimes since these states quickly relax out of this region if the system is flexible enough, as demonstrated by the current **C1** complex. A similar conclusion was drawn from our previous comparison of bidentate vs. tridentate coordination where the associated rigidity dictates the participation of the triplet states and their ability to stretch Fe–N bonds [14]. Longer ES lifetimes may be achieved by blocking the elongations of the Fe–N bonds [51] or through their substitution by more photoresistant bonds such as Fe–C ones [15,52,53,54].

In the case of **C2**, while the increase of the rigidity of the molecular scaffold could allow the improvement of the photophysical properties, the presence of six Fe–N bonds strongly diminishes the ligand-field splitting and hence strongly shortens the ES lifetime due to the stabilization of the metal centered quintet state that is actively participating in the relaxation. All in all, these contrasting observations clearly points to the subtle equilibrium between the different electronic and geometrical factors susceptible of altering the photophysical outcome of iron(II)-based complexes. The competition between the different factors needs to be fully considered when proceeding to rational molecular design strategy for improving the optical response.

## Figures and Tables

**Table 1:** Bond lengths and coordination angles of **C0** and **C1**.

Bond Length (Å)				Angles (°)			
C0		C1		C0		C1	
Fe1-N3	1.919(3)	Fe1-N1	2.035(11)	C1-Fe1-N3	79.5(1)	C1-Fe1-N1	87.7(5)
Fe–N8	1.930(3)	Fe1-N1′	2.035(11)	C11-Fe1-N3	79.2(1)	C12-Fe1-N1	88.5(4)
Fe1-C11	1.965(3)	Fe1-C12	1.981(11)	C1-Fe1-C11	158.0(2)	C12-Fe1-C1	176.1(5)
Fe1-C14	1.965(3)	Fe1-C1′	1.981(10)	C14-Fe1-N8	79.0(1)	C1′-Fe1-N1′	87.7(5)
Fe1-C1	1.966(3)	Fe1-C1	1.981(10)	C22-Fe1-N8	79.0(1)	C12′-Fe1-N1′	88.5(5)
Fe-C22	1.970(3)	Fe1-C12′	1.981(11)	C14-Fe1-C22	158.0(2)	C1′-Fe1-C12′	176.1(5)

**Table 2:** Bond lengths and coordination angles of **C2.**

Bond Length (Å)		Angles (°)	
Fe1-N2	1.961(2)	N2-Fe1-N3	90.36(9)
Fe1-N2′	1.957(2)	N3′-Fe1-N1	90.88(8)
Fe1-N1	1.997(2)	N3-Fe1-N1	177.58(8)
Fe1-N1′	1.997(2)	N2′-Fe1-N1′	90.15(9)
Fe1-N3	1.986(2)	N2′-Fe1-N3′	90.48(9)
Fe1-N3′	1.995(2)	N3′-Fe1-N1′	179.21(9)

**Table 3:** Photophysical and electrochemical properties of **C0**, **C1** and **C2**.

Complex	λabs-Max ^a^ (nm) ε(M^−1^.cm^−1^)	E_ox_ (Fe^III^/Fe^II^) ^b^ (V/SCE)	E_red1_ (V/SCE)	ΔE ^c^ (eV)
**C0**	393 (9000) 460 (15,900)	0.71 (rev)	−2.00 (irrev)	2.75
**C1**	525 (4800)	0.31 (rev)	−2.05 (irrev)	2.36
**C2**	575 (14,200)	0.96 (rev)	−1.35 (rev)	2.31

^a^ Measured in CH_3_CN at 25 °C. ^b^ First oxidation potential. Potentials are quoted vs (SCE). Under these conditions, E_1/2_ (Fc+/Fc) = 0.39 V/SCE. Recorded in CH_3_CN using Bu_4_N^+^,PF_6_^−^ (0.1 M) as supporting electrolyte at 100 mV.s^−1^. ^c^ Electrochemical band gap (ΔE = E_ox_ − E_red1_).

**Table 4:** Fe–N distances (in Å) corresponding to the structures displayed in Figure 8.

Fe–N	S_0_ min	S_1_ min	T_1_ min	Q_1_ min
**C1**				
**Fe–N1**	2.120	2.151	2.604	2.491
**Fe–N1′**	2.122	2.042	2.417	2.490
**C2**				
**Fe–N1**	2.013	2.003	2.281	2.190
**Fe–N2**	1.975	1.960	1.955	2.179
**Fe–N3**	2.013	2.019	2.283	2.189
**Fe–N1′**	2.013	2.010	2.121	2.188
**Fe–N2′**	1.976	1.973	1.937	2.179
**Fe–N3′**	2.013	2.024	2.120	2.191

## Supplementary Materials

The following are available online, Figures S1–S6 : ^1^H and ^13^C-NMR spectra of **L1**, **L2**, **C1** and **C2**, Figure S7: transient spectroscopy, quality of global fit for **C1**, Figure S8: transient spectroscopy, analysis of vibrational coherence in **C1**, Figure S9: transient spectroscopy, global fit results for **C2**, Figure S10: Computational data, Scan of one Fe-N bond distance of **C1** relaxing the T_1_ state, Tables S1–S15: Xray parameters for **C1** and **C2**, Table S16: transient spectroscopy: fit parameters for **C1**. CCDC No. are 199232 for **C1** and 1982173 for **C2**.

## References

[1] Hagfeldt A., Boschloo G., Sun L., Kloo L., Pettersson H. (2010). Dye-Sensitized Solar Cells. Chem. Rev..

[2] Vos J.G., Kelly J.M. (2006). Ruthenium polypyridyl chemistry; from basic research to applications and back again. Dalton Trans..

[3] Pastore M., Selloni A., Fantacci S., De Angelis F., Di Valentin C., Botti S., Cococcioni M. (2014). Electronic and Optical Properties of Dye-Sensitized TiO_2_ Interfaces. First Principles Approaches to Spectroscopic Properties of Complex Materials.

[4] Duchanois T., Etienne T., Cebrián C., Liu L., Monari A., Beley M., Assfeld X., Haacke S., Gros P.C. (2015). An Iron-Based Photosensitizer with Extended Excited-State Lifetime: Photophysical and Photovoltaic Properties: An Iron-Based Photosensitizer with Extended Excited-State Lifetime. Eur. J. Inorg. Chem..

[5] Marchini E., Darari M., Lazzarin L., Boaretto R., Argazzi R., Bignozzi C.A., Gros P.C., Caramori S. (2020). Recombination and regeneration dynamics in FeNHC(II)-sensitized solar cells. Chem. Commun..

[6] Creutz C., Chou M., Netzel T.L., Okumura M., Sutin N. (1980). Lifetimes, spectra, and quenching of the excited states of polypyridine complexes of iron(II), ruthenium(II), and osmium(II). J. Am. Chem. Soc..

[7] Gawelda W., Cannizzo A., Pham V.-T., van Mourik F., Bressler C., Chergui M. (2007). Ultrafast Nonadiabatic Dynamics of [Fe^II^(bpy)_3_]^2+^ in Solution. J. Am. Chem. Soc..

[8] Liu Y., Harlang T., Canton S.E., Chábera P., Suárez-Alcántara K., Fleckhaus A., Vithanage D.A., Göransson E., Corani A., Lomoth R. (2013). Towards longer-lived metal-to-ligand charge transfer states of iron(II) complexes: An N-heterocyclic carbene approach. Chem. Commun..

[9] Liu L., Duchanois T., Etienne T., Monari A., Beley M., Assfeld X., Haacke S., Gros P.C. (2016). A new record excited state ^3^ MLCT lifetime for metalorganic iron (II) complexes. Phys. Chem. Chem. Phys..

[10] Duchanois T., Liu L., Pastore M., Monari A., Cebrián C., Trolez Y., Darari M., Magra K., Francés-Monerris A., Domenichini E. (2018). NHC-Based Iron Sensitizers for DSSCs. Inorganics.

[11] Francés-Monerris A., Magra K., Darari M., Cebrián C., Beley M., Domenichini E., Haacke S., Pastore M., Assfeld X., Gros P.C. (2018). Synthesis and Computational Study of a Pyridylcarbene Fe(II) Complex: Unexpected Effects of *fac*/*mer* Isomerism in Metal-to-Ligand Triplet Potential Energy Surfaces. Inorg. Chem..

[12] Darari M., Domenichini E., Francés-Monerris A., Cebrián C., Magra K., Beley M., Pastore M., Monari A., Assfeld X., Haacke S. (2019). Iron(II) complexes with diazinyl-NHC ligands: Impact of π-deficiency of the azine core on photophysical properties. Dalton Trans..

[13] Magra K., Domenichini E., Francés-Monerris A., Cebrián C., Beley M., Darari M., Pastore M., Monari A., Assfeld X., Haacke S. (2019). Impact of the *fac*/*mer* Isomerism on the Excited-State Dynamics of Pyridyl-carbene Fe(II) Complexes. Inorg. Chem..

[14] Magra K., Darari M., Domenichini E., Francés-Monerris A., Cebrián C., Beley M., Pastore M., Monari A., Assfeld X., Haacke S. (2020). Photophysical Investigation of Iron(II) Complexes Bearing Bidentate Annulated Isomeric Pyridine-NHC Ligands. J. Phys. Chem. C.

[15] Chábera P., Kjaer K.S., Prakash O., Honarfar A., Liu Y., Fredin L.A., Harlang T.C.B., Lidin S., Uhlig J., Sundström V. (2018). Fe^II^ Hexa *N*-Heterocyclic Carbene Complex with a 528 ps Metal-to-Ligand Charge-Transfer Excited-State Lifetime. J. Phys. Chem. Lett..

[16] Braun J.D., Lozada I.B., Kolodziej C., Burda C., Newman K.M.E., van Lierop J., Davis R.L., Herbert D.E. (2019). Iron(II) coordination complexes with panchromatic absorption and nanosecond charge-transfer excited state lifetimes. Nat. Chem..

[17] Lindh L., Chábera P., Rosemann N.W., Uhlig J., Wärnmark K., Yartsev A., Sundström V., Persson P. (2020). Photophysics and Photochemistry of Iron Carbene Complexes for Solar Energy Conversion and Photocatalysis. Catalysts.

[18] Abrahamsson M., Jäger M., Österman T., Eriksson L., Persson P., Becker H.-C., Johansson O., Hammarström L. (2006). A 3.0 μs Room Temperature Excited State Lifetime of a Bistridentate Ru^II^—Polypyridine Complex for Rod-like Molecular Arrays. J. Am. Chem. Soc..

[19] Hu Y.-Z., Wilson M.H., Zong R., Bonnefous C., McMillin D.R., Thummel R.P. (2005). A luminescent Pt(II) complex with a terpyridine-like ligand involving a six-membered chelate ring. Dalton Trans..

[20] Vezzu D.A.K., Ravindranathan D., Garner A.W., Bartolotti L., Smith M.E., Boyle P.D., Huo S. (2011). Highly Luminescent Tridentate N^∧^C*N Platinum(II) Complexes Featured in Fused Five–Six-Membered Metallacycle and Diminishing Concentration Quenching. Inorg. Chem..

[21] Mengel A.K.C., Förster C., Breivogel A., Mack K., Ochsmann J.R., Laquai F., Ksenofontov V., Heinze K. (2015). A Heteroleptic Push-Pull Substituted Iron(II) Bis(tridentate) Complex with Low-Energy Charge-Transfer States. Chem. Eur. J..

[22] Kjær K.S., Kaul N., Prakash O., Chábera P., Rosemann N.W., Honarfar A., Gordivska O., Fredin L.A., Bergquist K.-E., Häggström L. (2019). Luminescence and reactivity of a charge-transfer excited iron complex with nanosecond lifetime. Science.

[23] Sauvage J.P., Collin J.P., Chambron J.C., Guillerez S., Coudret C., Balzani V., Barigelletti F., De Cola L., Flamigni L. (1994). Ruthenium(II) and Osmium(II) Bis(terpyridine) Complexes in Covalently-Linked Multicomponent Systems: Synthesis, Electrochemical Behavior, Absorption Spectra, and Photochemical and Photophysical Properties. Chem. Rev..

[24] Campagna S., Puntoriero F., Nastasi F., Bergamini G., Balzani V., Balzani V., Campagna S. (2007). Photochemistry and Photophysics of Coordination Compounds: Ruthenium. Photochemistry and Photophysics of Coordination Compounds I.

[25] Rillema D.P., Jones D.S. (1979). Structure of tris(2,2′-bipyridyl)ruthenium(II) hexafluorophosphate, [Ru(bipy)_3_][PF_6_]_2_; X-ray crystallographic determination. J. Chem. Soc. Chem. Commun..

[26] Medlycott E.A., Hanan G.S. (2005). Designing tridentate ligands for ruthenium(II) complexes with prolonged room temperature luminescence lifetimes. Chem. Soc. Rev..

[27] Dinda J., Liatard S., Chauvin J., Jouvenot D., Loiseau F. (2011). Electronic and geometrical manipulation of the excited state of bis-terdentate homo- and heteroleptic ruthenium complexes. Dalton Trans..

[28] Otto S., Grabolle M., Förster C., Kreitner C., Resch-Genger U., Heinze K. (2015). [Cr(ddpd)_2_]^3+^: A Molecular, Water-Soluble, Highly NIR-Emissive Ruby Analogue. Angew. Chem. Int. Ed..

[29] Otto S., Scholz N., Behnke T., Resch-Genger U., Heinze K. (2017). Thermo-Chromium: A Contactless Optical Molecular Thermometer. Chem. Eur. J..

[30] Jamula L.L., Brown A.M., Guo D., McCusker J.K. (2014). Synthesis and Characterization of a High-Symmetry Ferrous Polypyridyl Complex: Approaching the ^5^T_2_/^3^T_1_ Crossing Point for Fe^II^. Inorg. Chem..

[31] Gründemann S., Albrecht M., Loch J.A., Faller J.W., Crabtree R.H. (2001). Tridentate Carbene CCC and CNC Pincer Palladium(II) Complexes: Structure, Fluxionality, and Catalytic Activity. Organometallics.

[32] Slavov C., Hartmann H., Wachtveitl J. (2015). Implementation and Evaluation of Data Analysis Strategies for Time-Resolved Optical Spectroscopy. Anal. Chem..

[33] Snellenburg J.J., Laptenok S.P., Seger R., Mullen K.M., Stokkum I.H.M. (2012). van Glotaran: A *Java* -Based Graphical User Interface for the *R* Package TIMP. J. Stat. Softw..

[34] Tatsuno H., Kjær K.S., Kunnus K., Harlang T.C.B., Timm C., Guo M., Chàbera P., Fredin L.A., Hartsock R.W., Reinhard M.E. (2020). Hot Branching Dynamics in a Light-Harvesting Iron Carbene Complex Revealed by Ultrafast X-ray Emission Spectroscopy. Angew. Chem. Int. Ed..

[35] Kunnus K., Vacher M., Harlang T.C.B., Kjær K.S., Haldrup K., Biasin E., van Driel T.B., Pápai M., Chabera P., Liu Y. (2020). Vibrational wavepacket dynamics in Fe carbene photosensitizer determined with femtosecond X-ray emission and scattering. Nat. Commun..

[36] Cannizzo A., Milne C.J., Consani C., Gawelda W., Bressler C., van Mourik F., Chergui M. (2010). Light-induced spin crossover in Fe(II)-based complexes: The full photocycle unraveled by ultrafast optical and X-ray spectroscopies. Coord. Chem. Rev..

[37] Consani C., Prémont-Schwarz M., ElNahhas A., Bressler C., van Mourik F., Cannizzo A., Chergui M. (2009). Vibrational Coherences and Relaxation in the High-Spin State of Aqueous [Fe^II^(bpy)_3_]^2+^. Angew. Chem. Int. Ed..

[38] Wolf M.M.N., Groß R., Schumann C., Wolny J.A., Schünemann V., Døssing A., Paulsen H., McGarvey J.J., Diller R. (2008). Sub-picosecond time resolved infrared spectroscopy of high-spin state formation in Fe(II) spin crossover complexes. Phys. Chem. Chem. Phys..

[39] Francés-Monerris A., Gros P.C., Assfeld X., Monari A., Pastore M. (2019). Toward Luminescent Iron Complexes: Unravelling the Photophysics by Computing Potential Energy Surfaces. ChemPhotoChem.

[40] Dolomanov O.V., Bourhis L.J., Gildea R.J., Howard J.A.K., Puschmann H. (2009). OLEX2: A complete structure solution, refinement and analysis program. J. Appl. Crystallogr..

[41] Sheldrick G.M. (2008). A short history of SHELX. Acta Crystallogr. A.

[42] Francés-Monerris A., Gros P.C., Pastore M., Assfeld X., Monari A. (2019). Photophysical properties of bichromophoric Fe(II) complexes bearing an aromatic electron acceptor. Theor. Chem. Acc..

[43] Frisch M.J., Trucks G.W., Schlegel H.B., Scuseria G.E., Robb M.A., Cheeseman J.R., Scalmani G., Barone V., Petersson G.A., Nakatsuji H. (2016). Gaussian 16, Revision C.01. https://gaussian.com/citation/.

[44] Boese A.D., Handy N.C. (2001). A new parametrization of exchange–correlation generalized gradient approximation functionals. J. Chem. Phys..

[45] Hirata S., Head-Gordon M. (1999). Time-dependent density functional theory within the Tamm–Dancoff approximation. Chem. Phys. Lett..

[46] Grimme S., Antony J., Ehrlich S., Krieg H. (2010). A consistent and accurate ab initio parametrization of density functional dispersion correction (DFT-D) for the 94 elements H-Pu. J. Chem. Phys..

[47] Martin R.L. (2003). Natural transition orbitals. J. Chem. Phys..

[48] Etienne T., Assfeld X., Monari A. (2014). Toward a Quantitative Assessment of Electronic Transitions’ Charge-Transfer Character. J. Chem. Theory Comput..

[49] Etienne T., Assfeld X., Monari A. (2014). New Insight into the Topology of Excited States through Detachment/Attachment Density Matrices-Based Centroids of Charge. J. Chem. Theory Comput..

[50] Pápai M., Vankó G., Rozgonyi T., Penfold T.J. (2016). High-Efficiency Iron Photosensitizer Explained with Quantum Wavepacket Dynamics. J. Phys. Chem. Lett..

[51] Paulus B.C., Adelman S.L., Jamula L.L., McCusker J.K. (2020). Leveraging excited-state coherence for synthetic control of ultrafast dynamics. Nature.

[52] Bauer M., Steube J., Päpcke A., Bokareva O., Reuter T., Demeshko S., Schoch R., Hohloch S., Meyer F., Heinze K. (2020). Janus-type dual emission of a Cyclometalated Iron(III) complex.

[53] Steube J., Burkhardt L., Päpcke A., Moll J., Zimmer P., Schoch R., Wölper C., Heinze K., Lochbrunner S., Bauer M. (2019). Excited-State Kinetics of an Air-Stable Cyclometalated Iron(II) Complex. Chem. Eur. J..

[54] Tang Z., Chang X.-Y., Wan Q., Wang J., Ma C., Law K.-C., Liu Y., Che C.-M. (2020). Bis(tridentate) Iron(II) Complexes with a Cyclometalating Unit: Photophysical Property Enhancement with Combinatorial Strong Ligand Field Effect. Organometallics.

