# Supplementary material for: Towards Iron(II) Complexes with Octahedral Geometry: Synthesis, Structure and Photophysical Properties
Source: Molecules. 2020 Dec 17;25(24):5991. doi: 10.3390/molecules25245991 (PMC7767130; doi:10.3390/molecules25245991)
Supplement: Supplementary file 1 [file molecules-25-05991-s001.pdf]

SUPPORTING INFORMATION

Towards Fe(II) complexes with octahedral geometry: synthesis, structure and photophysical properties

Mohamed Darari [1], Antonio Francés-Monerris [2,3], Bogdan Marekha [4], Abdelatif Doudouh [5], Emmanuel Wenger [5], Antonio Monari [2], Stefan Haacke [6] and Philippe C. Gros [1,*]

[1] Université de Lorraine, CNRS, L2CM, , F-54000 Nancy, France; mohamed.darari@univ-lorraine.fr (M.D.) ; philippe.gros@univ-lorraine.fr (P.C.G.)
[2] Université de Lorraine, CNRS, LPCT, F-54000 Nancy, France; antonio.frances@univ-lorraine.fr (A.F.-M.); Antonio.Monari@univ-lorraine.fr (A.M.)
[3] Departament de Química Física, Universitat de València, 46100 Burjassot, Spain
[4] Max-Planck-Institute for Medical Research, 69120 Heidelberg, Germany; bogdan.a.marekha@gmail.com (B.M.)
[5] Université de Lorraine, CNRS, CRM2, F-54000 Nancy, France; abdelatif.doudouh@univ-lorraine.fr (A.D.); emmanuel.wenger@univ-lorraine.fr (E.W.)
[6] Université de Strasbourg, CNRS, IPCMS, F-67034 Strasbourg, France; stefan.haacke@ipcms.unistra.fr) (S.H.)

* Correspondence: philippe.gros@univ-lorraine.fr

TABLE OF CONTENTS

NMR Spectra of ligands and complexes 2
Xray data 5
Transient spectroscopy details 20
Additional computational data 23
References 23

NMR Spectra of ligands and complexes

Figure S1. ^{1}H NMR of **L1** in in CD_3CN

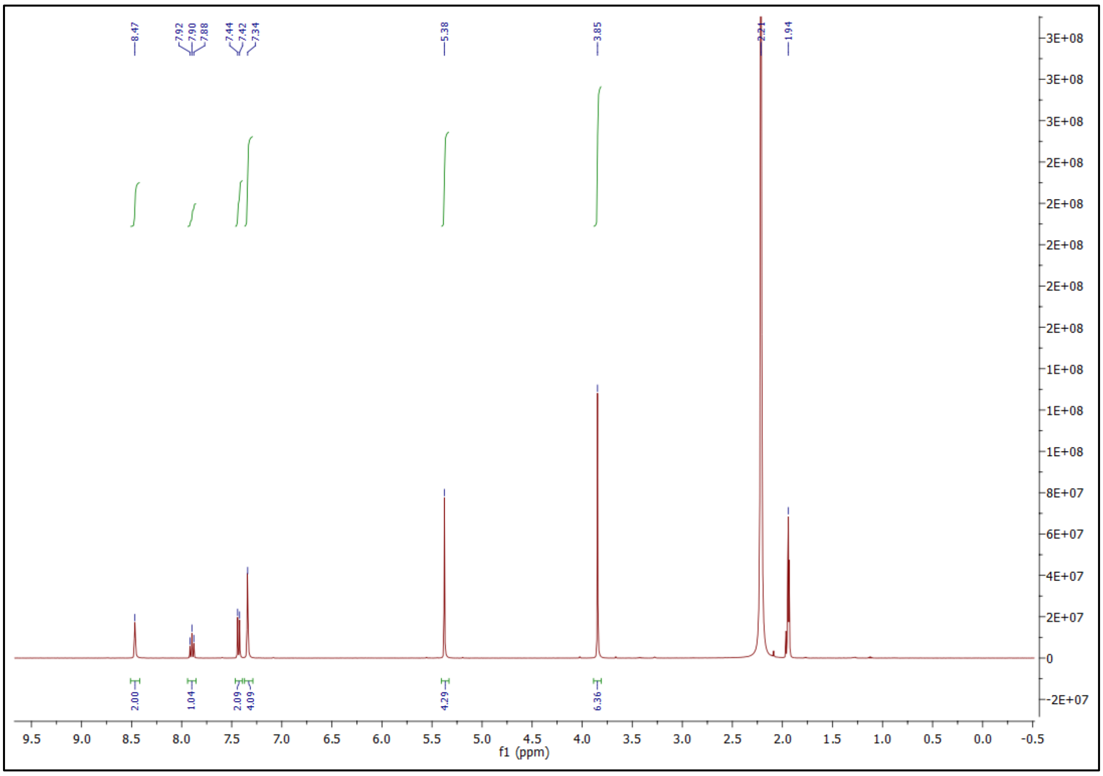

Figure S2. ^{1}H NMR of **dqp** in $CDCl_3$

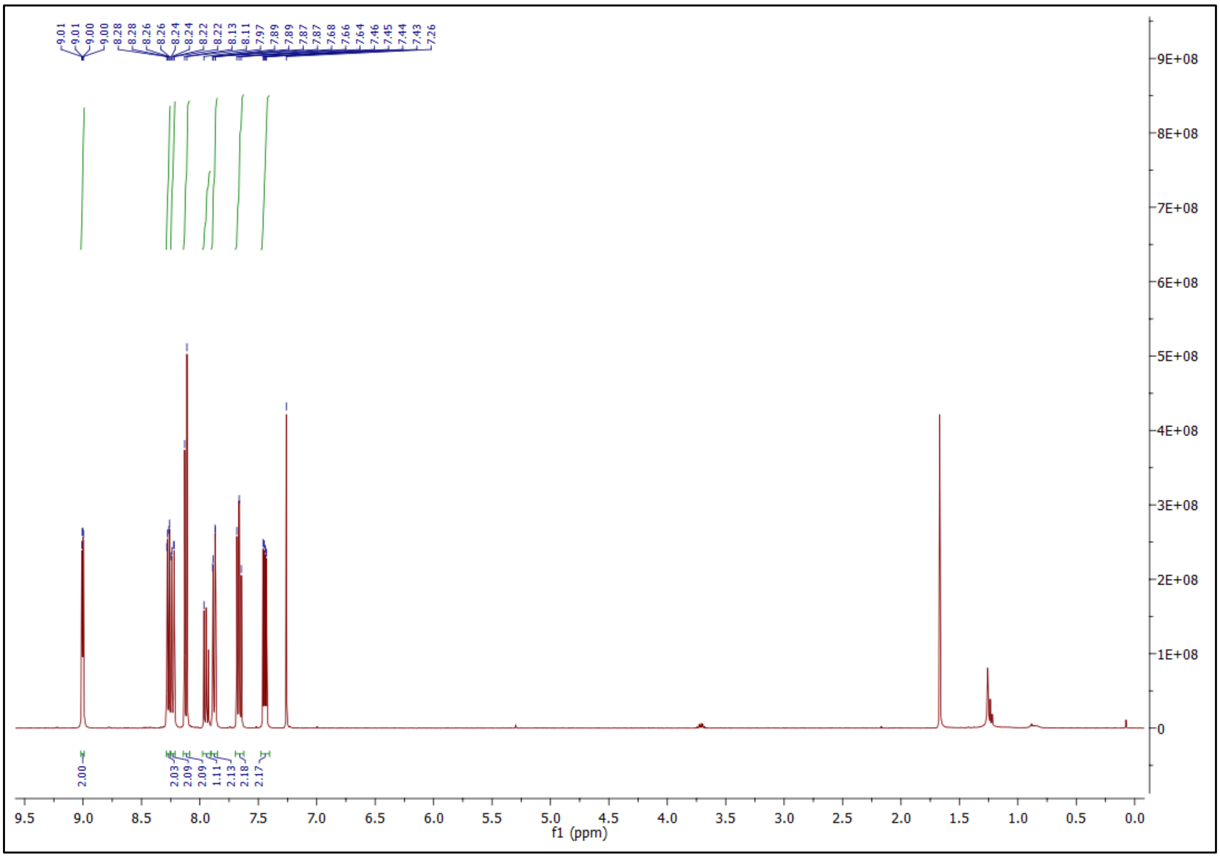

Figure S3. ^{1}H NMR of **C1** in CD_3CN

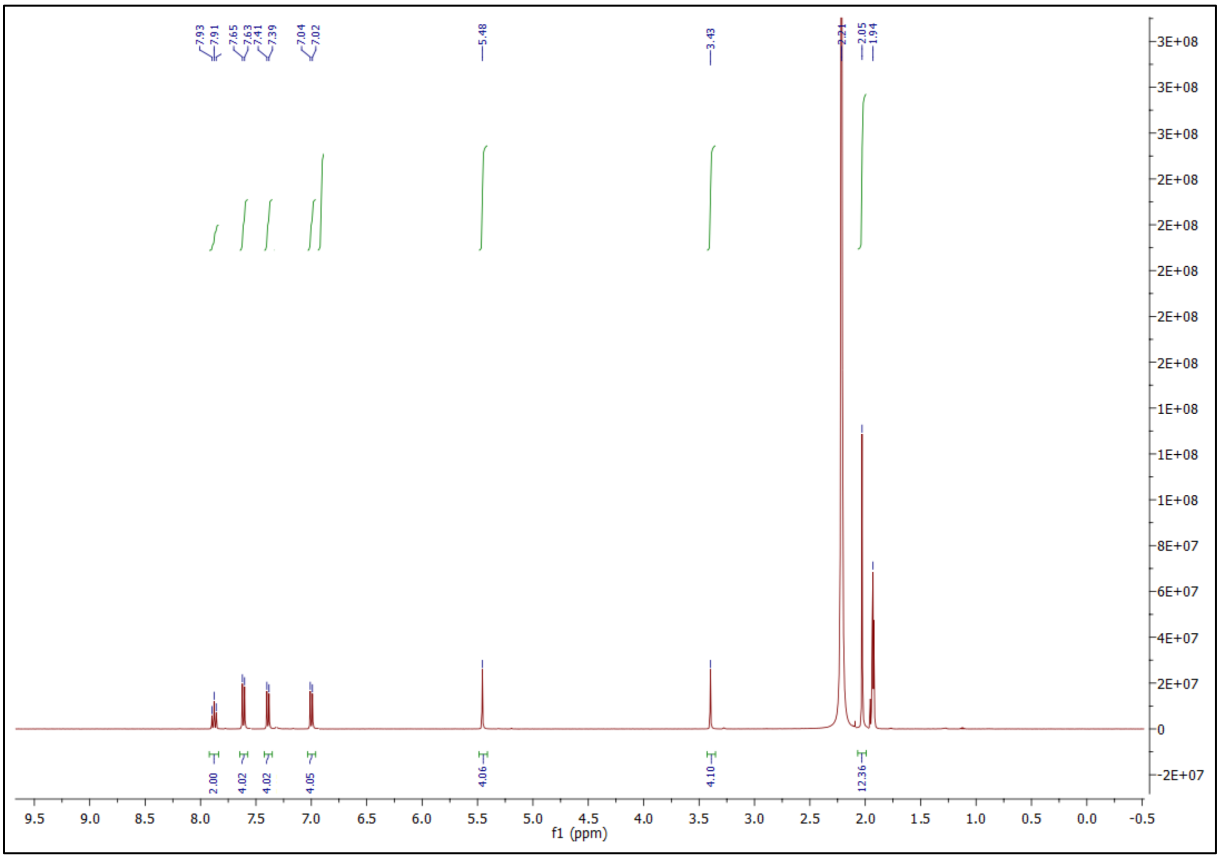

Figure S4. ^{13}C NMR of **C1** in CD_3CN

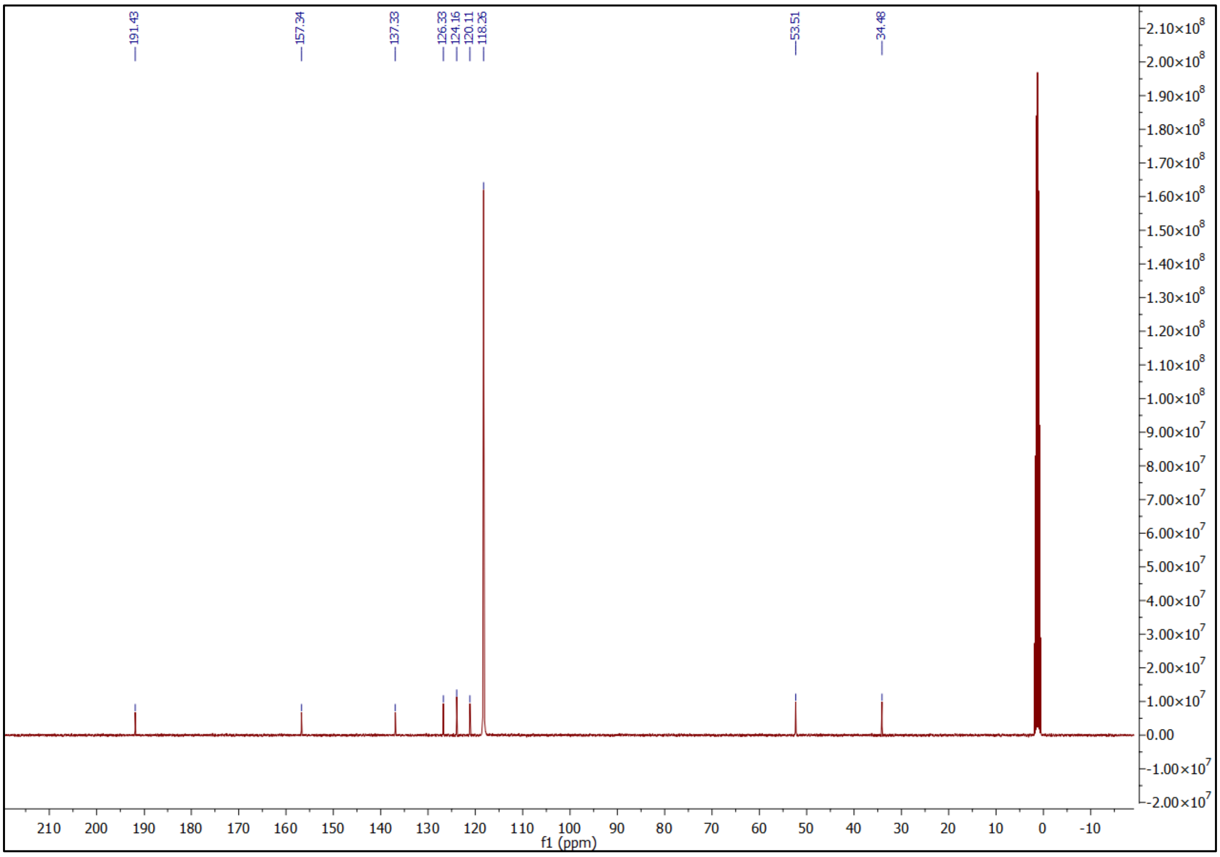

Figure S5. ^{1}H NMR of **C2** in CD_3CN

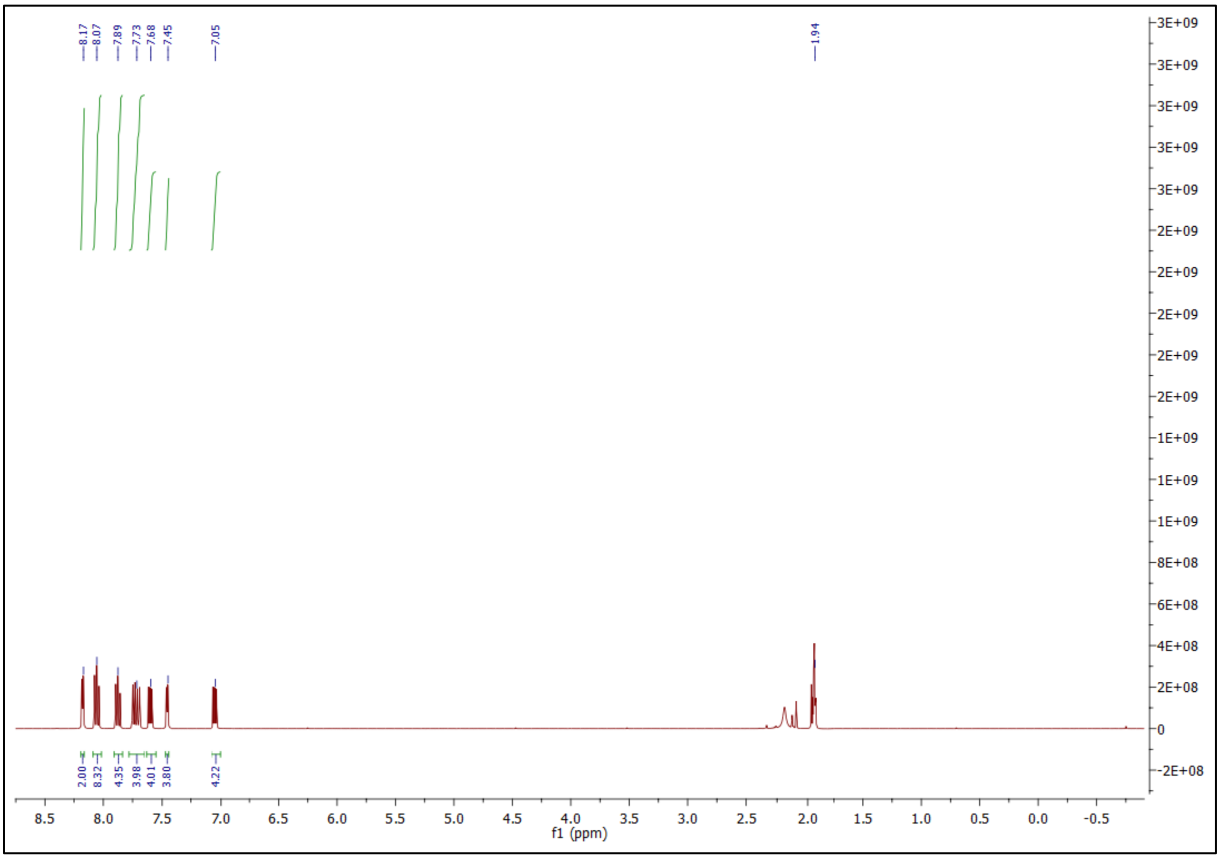

Figure S6. ^{13}C NMR of **C2** in acetonitrile-d3

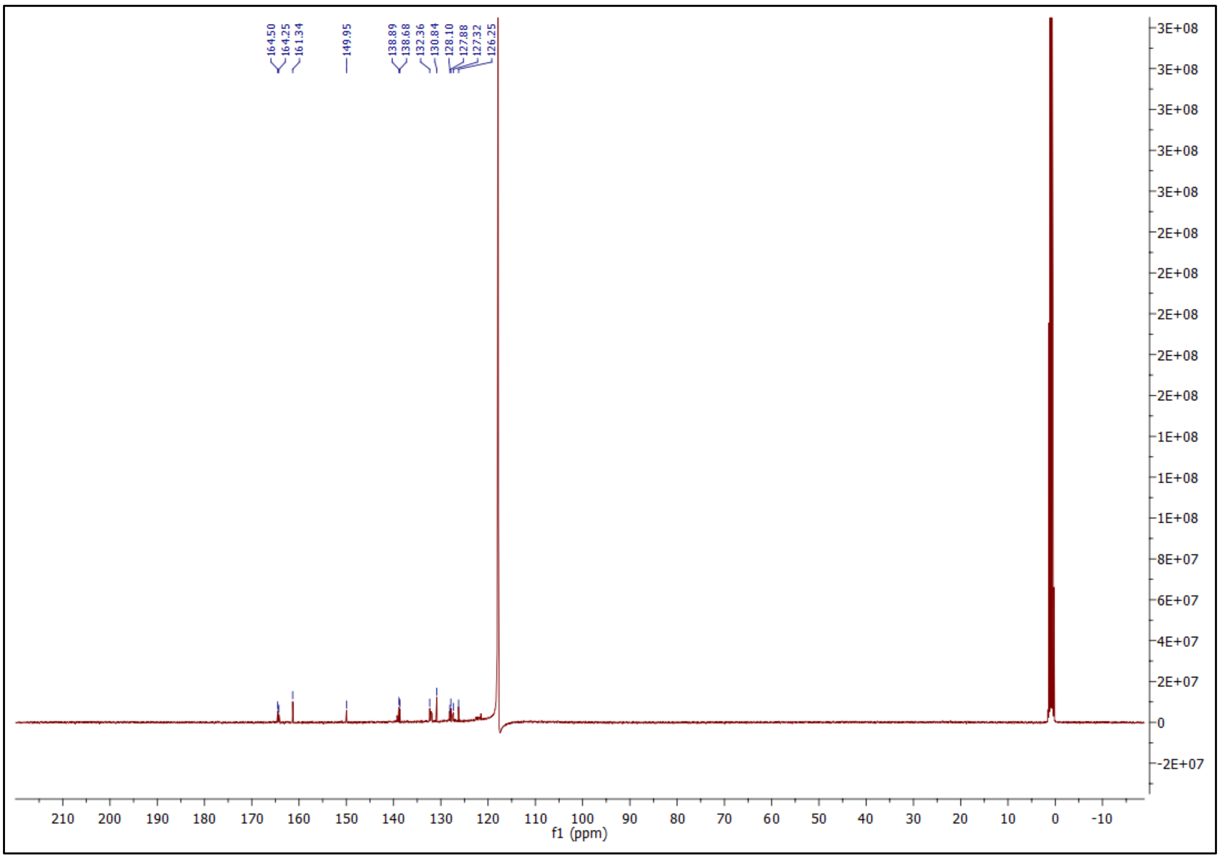

Xray data
Complex C1

Table S1 Crystal data and structure refinement for C1.

Identification code	C1
Empirical formula	$C_{30}H_{34}F_{12}FeN_{10}P_2$
Formula weight	880.46
Temperature/K	100.0(1)
Crystal system	monoclinic
Space group	P2/c
a/Å	21.392(2)
b/Å	9.1122(5)
c/Å	20.278(2)
α/°	90
β/°	117.306(14)
γ/°	90
Volume/Å^3	3512.4(7)
Z	4
ρ_{calc}g/cm^3	1.665
μ/mm^{-1}	0.625
F(000)	1792.0
Crystal size/mm^3	0.147 × 0.139 × 0.098
Radiation	MoKα (λ = 0.71073)
2Θ range for data collection/°	3.76 to 52.742
Index ranges	-26 ≤ h ≤ 26, -11 ≤ k ≤ 11, -25 ≤ l ≤ 25
Reflections collected	47631
Independent reflections	7182 [R_{int} = 0.0912, R_{sigma} = 0.0581]
Data/restraints/parameters	7182/0/515
Goodness-of-fit on F^2	1.023
Final R indexes [I>=2σ (I)]	R_1 = 0.0958, wR_2 = 0.2453
Final R indexes [all data]	R_1 = 0.1196, wR_2 = 0.2724
Largest diff. peak/hole / e Å^{-3}	1.51/-1.02

Table S2 Fractional Atomic Coordinates (×10^4) and Equivalent Isotropic Displacement Parameters (Å^2×10^3) for C1. U_{eq} is defined as 1/3 of of the trace of the orthogonalised U_{IJ} tensor.

Atom	*x*	*y*	*z*	U(eq)
Fe1	0	9192.6(17)	2500	29.5(4)
Fe1B	-5000	10249.4(16)	-2500	29.3(4)
P2	-2094.3(13)	6171(3)	-1625.7(15)	43.6(6)
F1B	-2188(3)	7877(7)	-1528(5)	72(2)
N4	-557(4)	7441(8)	1030(4)	39.8(16)
N1B	-3972(3)	10292(7)	-1719(4)	30.0(14)
N5B	-4684(4)	7671(7)	-1533(4)	33.7(15)
N5	402(4)	6614(7)	1890(4)	39.2(16)
N4B	-5701(4)	8399(8)	-1740(4)	38.4(16)
N2B	-4246(4)	12704(7)	-2750(4)	38.7(17)

F3B	-1811(5)	6523(8)	-2205(4)	77(2)
N3B	-4917(4)	11806(7)	-3807(4)	39.9(16)
F4B	-2880(3)	6095(8)	-2240(5)	88(3)
N1	1061(4)	9126(7)	2855(4)	35.8(16)
N2	679(4)	11688(7)	3480(4)	38.7(16)
F2B	-1992(4)	4488(7)	-1724(5)	82(2)
F5B	-2352(7)	5829(10)	-1025(6)	117(4)
C12	-87(5)	7704(8)	1754(4)	32.5(17)
F6B	-1300(4)	6338(10)	-1003(5)	101(3)
N3	-98(4)	10881(8)	3763(4)	41.1(17)
C6B	-3586(4)	11557(8)	-1541(5)	35.5(18)
C12B	-5173(4)	8721(8)	-1914(4)	31.5(17)
C8B	-2607(5)	10446(10)	-553(6)	53(3)
C1B	-4765(4)	11676(8)	-3089(5)	34.6(18)
C10	1413(4)	7868(9)	2890(5)	36.4(18)
C2B	-5443(5)	10975(10)	-4435(5)	41(2)
C9	2132(5)	7766(11)	3179(6)	48(2)
C3B	-4491(5)	12851(10)	-3920(6)	47(2)
C11B	-3988(4)	7613(9)	-1521(5)	37.3(18)
C11	1009(5)	6442(9)	2618(6)	43(2)
C1	158(4)	10674(9)	3278(5)	35.8(19)
C10B	-3634(5)	9097(8)	-1317(5)	35.1(17)
C7B	-2908(5)	11663(9)	-974(6)	46(2)
C15B	-6363(5)	9189(10)	-2007(6)	47(2)
C9B	-2963(5)	9142(10)	-722(6)	45(2)
C4	772(6)	12475(10)	4093(6)	52(2)
C7	2195(5)	10296(10)	3457(6)	51(2)
C15	-1196(5)	8269(10)	583(5)	44(2)
C14B	-5545(6)	7175(10)	-1283(6)	51(2)
C13	250(5)	5748(11)	1302(6)	48(2)
C2	-704(5)	10126(11)	3776(7)	52(2)
C14	-349(5)	6251(10)	747(5)	45(2)
C4B	-4071(5)	13412(9)	-3255(6)	43(2)
C5	1129(5)	11799(10)	3106(6)	43(2)
C8	2545(5)	9001(11)	3492(7)	55(3)
C6	1476(5)	10348(9)	3135(5)	41(2)
C13B	-4906(6)	6725(10)	-1147(5)	47(2)
C3	267(6)	11986(11)	4267(6)	54(3)
C5B	-3920(4)	12951(8)	-1956(5)	36.4(17)
P1A	-3177(3)	5399(7)	-4605(3)	41.1(18)
F6A	-2331(3)	5184(12)	-4343(6)	69(3)
F4A	-3025(4)	5328(9)	-3733(3)	56(2)
F3A	-4023(3)	5614(9)	-4868(5)	66(3)
F1A	-3329(5)	5469(14)	-5477(4)	55(4)
F2A	-3284(4)	3606(7)	-4671(5)	52(3)
F5A	-3070(5)	7191(7)	-4540(6)	62(4)
P1A'	-2961(6)	5470(10)	-4568(5)	41.1(18)
F4A'	-2370(7)	6253(18)	-4680(8)	69(3)
F3A'	-2482(7)	5441(13)	-3703(7)	56(2)
F1A'	-3602(8)	4752(16)	-4468(8)	66(3)
F5A'	-2731(12)	3944(15)	-4676(8)	87(6)
F2A'	-3282(11)	6983(17)	-4482(11)	62(4)
F6A'	-3508(11)	5520(20)	-5427(9)	55(4)

Table S3 Anisotropic Displacement Parameters (Å^2×10^3) for C1. The Anisotropic displacement factor exponent takes the form: $-2\pi^2[h^2a^{*2}U_{11}+2hka^*b^*U_{12}+…]$.

Atom	U_{11}	U_{22}	U_{33}	U_{23}	U_{13}	U_{12}
Fe1	28.2(7)	19.8(8)	37.1(9)	0	11.9(7)	0
Fe1B	32.5(8)	17.5(7)	36.7(9)	0	14.9(7)	0
P2	43.6(13)	40.4(13)	46.5(13)	-6.6(10)	20.5(11)	1.6(10)
F1B	52(3)	44(3)	116(6)	-23(3)	34(4)	0(3)
N4	36(4)	30(3)	48(4)	-4(3)	15(3)	4(3)
N1B	32(3)	22(3)	35(4)	2(3)	14(3)	0(3)
N5B	40(4)	21(3)	34(3)	0(3)	12(3)	-5(3)
N5	39(4)	24(3)	54(4)	0(3)	20(3)	5(3)
N4B	56(4)	32(4)	36(4)	-6(3)	29(3)	-9(3)
N2B	39(4)	21(3)	51(4)	5(3)	16(3)	3(3)
F3B	105(6)	68(4)	78(5)	-19(4)	58(4)	-26(4)
N3B	40(4)	27(3)	55(5)	5(3)	24(4)	3(3)
F4B	41(3)	63(4)	125(7)	-16(4)	8(4)	-6(3)
N1	36(4)	23(3)	44(4)	0(3)	14(3)	-1(3)
N2	44(4)	21(3)	47(4)	-3(3)	18(3)	1(3)
F2B	98(6)	35(3)	124(6)	0(4)	60(5)	12(3)
F5B	192(11)	86(6)	130(8)	23(6)	122(8)	34(6)
C12	43(5)	18(3)	35(4)	-1(3)	16(3)	1(3)
F6B	59(4)	109(6)	87(5)	-44(5)	-8(4)	35(4)
N3	52(5)	31(4)	37(4)	0(3)	17(3)	2(3)
C6B	35(4)	20(4)	52(5)	5(3)	20(4)	-1(3)
C12B	40(4)	20(3)	35(4)	-5(3)	17(3)	-4(3)
C8B	36(5)	39(5)	64(6)	-1(4)	6(4)	8(4)
C1B	34(4)	25(4)	46(5)	6(3)	18(4)	5(3)
C10	34(4)	30(4)	44(5)	0(3)	16(3)	1(3)
C2B	53(5)	36(4)	38(4)	4(4)	23(4)	9(4)
C9	38(5)	48(5)	56(6)	17(5)	18(4)	13(4)
C3B	57(6)	28(4)	65(6)	13(4)	37(5)	5(4)
C11B	36(4)	23(4)	44(5)	5(3)	11(4)	6(3)
C11	40(4)	22(4)	59(6)	0(4)	17(4)	4(3)
C1	39(5)	24(4)	36(4)	-5(3)	10(3)	4(3)
C10B	41(4)	21(4)	42(4)	2(3)	18(4)	5(3)
C7B	38(5)	25(4)	65(6)	-11(4)	15(4)	-3(3)
C15B	60(6)	29(4)	74(7)	-14(4)	48(5)	-12(4)
C9B	44(5)	29(4)	52(5)	2(4)	13(4)	10(4)
C4	54(6)	27(4)	64(6)	-9(4)	17(5)	-1(4)
C7	45(5)	33(5)	72(7)	-2(4)	24(5)	-11(4)
C15	47(5)	31(4)	42(5)	0(4)	9(4)	4(4)
C14B	69(7)	36(5)	61(6)	-1(4)	41(5)	-19(5)
C13	44(5)	39(5)	53(6)	-6(4)	15(4)	15(4)
C2	46(5)	39(5)	76(7)	8(5)	32(5)	3(4)
C14	51(5)	36(5)	47(5)	-18(4)	20(4)	-3(4)
C4B	45(5)	22(4)	65(6)	13(4)	26(4)	8(4)
C5	46(5)	30(4)	56(6)	-12(4)	27(4)	-10(4)
C8	31(4)	46(6)	79(7)	15(5)	17(4)	-2(4)
C6	47(5)	29(4)	47(5)	-6(4)	21(4)	-4(4)
C13B	63(6)	34(4)	43(5)	6(4)	25(5)	-5(4)
C3	74(7)	35(5)	58(6)	-11(4)	36(5)	8(5)
C5B	36(4)	23(4)	46(5)	3(4)	16(4)	-4(3)
P1A	55(5)	28.8(13)	37.4(15)	-2.8(11)	20(2)	-1(2)
F6A	43(4)	83(7)	71(7)	-4(5)	18(5)	-5(5)
F4A	68(5)	47(5)	39(4)	-7(3)	11(4)	3(5)

F3A	72(7)	60(6)	64(6)	-26(5)	29(5)	-9(4)
F1A	72(9)	49(4)	55(4)	-7(3)	39(4)	-31(5)
F2A	65(7)	26(5)	59(7)	-4(5)	23(6)	4(5)
F5A	95(10)	17(4)	63(5)	-4(3)	28(6)	-28(5)
P1A'	55(5)	28.8(13)	37.4(15)	-2.8(11)	20(2)	-1(2)
F4A'	43(4)	83(7)	71(7)	-4(5)	18(5)	-5(5)
F3A'	68(5)	47(5)	39(4)	-7(3)	11(4)	3(5)
F1A'	72(7)	60(6)	64(6)	-26(5)	29(5)	-9(4)
F5A'	156(18)	30(7)	46(7)	-4(6)	22(10)	19(9)
F2A'	95(10)	17(4)	63(5)	-4(3)	28(6)	-28(5)
F6A'	72(9)	49(4)	55(4)	-7(3)	39(4)	-31(5)

Table S4 Bond Lengths for C1.

Atom	Atom	Length/Å	Atom	Atom	Length/Å
Fe1	N1	2.043(7)	N3B	C3B	1.406(12)
Fe1	N1[1]	2.043(7)	N1	C10	1.356(11)
Fe1	C12	1.976(8)	N1	C6	1.373(11)
Fe1	C12[1]	1.976(8)	N2	C1	1.358(11)
Fe1	C1[1]	1.984(8)	N2	C4	1.368(13)
Fe1	C1	1.984(8)	N2	C5	1.476(12)
Fe1B	N1B	2.038(6)	N3	C1	1.340(12)
Fe1B	N1B[2]	2.038(6)	N3	C2	1.480(13)
Fe1B	C12B[2]	1.972(8)	N3	C3	1.391(12)
Fe1B	C12B	1.972(8)	C6B	C7B	1.381(13)
Fe1B	C1B[2]	1.980(8)	C6B	C5B	1.510(11)
Fe1B	C1B	1.980(8)	C8B	C7B	1.367(13)
P2	F1B	1.592(6)	C8B	C9B	1.367(13)
P2	F3B	1.583(8)	C10	C9	1.376(12)
P2	F4B	1.569(7)	C10	C11	1.517(12)
P2	F2B	1.574(6)	C9	C8	1.391(15)
P2	F5B	1.581(9)	C3B	C4B	1.333(15)
P2	F6B	1.592(7)	C11B	C10B	1.511(11)
N4	C12	1.367(11)	C10B	C9B	1.389(12)
N4	C15	1.458(11)	C4	C3	1.358(16)
N4	C14	1.392(11)	C7	C8	1.382(14)
N1B	C6B	1.366(10)	C7	C6	1.369(13)
N1B	C10B	1.354(10)	C14B	C13B	1.329(15)
N5B	C12B	1.365(10)	C13	C14	1.340(13)
N5B	C11B	1.479(12)	C5	C6	1.505(12)
N5B	C13B	1.386(11)	P1A	F6A	1.6466
N5	C12	1.376(11)	P1A	F4A	1.6459
N5	C11	1.461(12)	P1A	F3A	1.6470
N5	C13	1.339(12)	P1A	F1A	1.6457
N4B	C12B	1.363(11)	P1A	F2A	1.6467
N4B	C15B	1.453(13)	P1A	F5A	1.6465
N4B	C14B	1.388(12)	P1A'	F4A'	1.556(18)
N2B	C1B	1.371(11)	P1A'	F3A'	1.574(15)
N2B	C4B	1.397(12)	P1A'	F1A'	1.613(17)
N2B	C5B	1.450(12)	P1A'	F5A'	1.525(16)
N3B	C1B	1.345(12)	P1A'	F2A'	1.585(18)

N3B	C2B	1.465(12)	P1A'	F6A'	1.591(17)

Table S5 Bond Angles for C1.

Atom	Atom	Atom	Angle/°	Atom	Atom	Atom	Angle/°
N1^1	Fe1	N1	176.6(4)	N5	C12	Fe1	123.1(6)
C12	Fe1	N1^1	90.6(3)	C1	N3	C2	127.1(8)
C12	Fe1	N1	87.1(3)	C1	N3	C3	111.2(8)
C12^1	Fe1	N1	90.6(3)	C3	N3	C2	121.6(9)
C12^1	Fe1	N1^1	87.1(3)	N1B	C6B	C7B	123.7(7)
C12	Fe1	C12^1	93.3(4)	N1B	C6B	C5B	119.3(7)
C12	Fe1	C1	176.1(3)	C7B	C6B	C5B	117.0(7)
C12^1	Fe1	C1^1	176.1(3)	N5B	C12B	Fe1B	122.0(6)
C12	Fe1	C1^1	86.4(3)	N4B	C12B	Fe1B	135.7(6)
C12^1	Fe1	C1	86.4(3)	N4B	C12B	N5B	102.3(7)
C1	Fe1	N1	89.1(3)	C7B	C8B	C9B	120.0(9)
C1^1	Fe1	N1^1	89.1(3)	N2B	C1B	Fe1B	121.2(6)
C1	Fe1	N1^1	93.2(3)	N3B	C1B	Fe1B	135.2(6)
C1^1	Fe1	N1	93.2(3)	N3B	C1B	N2B	102.7(7)
C1	Fe1	C1^1	94.3(5)	N1	C10	C9	124.4(8)
N1B^2	Fe1B	N1B	177.8(4)	N1	C10	C11	119.7(7)
C12B^2	Fe1B	N1B^2	88.8(3)	C9	C10	C11	115.9(8)
C12B	Fe1B	N1B	88.8(3)	C10	C9	C8	119.5(9)
C12B	Fe1B	N1B^2	92.8(3)	C4B	C3B	N3B	106.3(9)
C12B^2	Fe1B	N1B	92.8(3)	N5B	C11B	C10B	110.3(7)
C12B	Fe1B	C12B^2	90.2(4)	N5	C11	C10	111.7(7)
C12B^2	Fe1B	C1B	86.0(3)	N2	C1	Fe1	121.3(7)
C12B	Fe1B	C1B	175.2(3)	N3	C1	Fe1	134.6(6)
C12B	Fe1B	C1B^2	86.0(3)	N3	C1	N2	103.8(7)
C12B^2	Fe1B	C1B^2	175.2(3)	N1B	C10B	C11B	119.6(7)
C1B^2	Fe1B	N1B	90.0(3)	N1B	C10B	C9B	123.5(8)
C1B	Fe1B	N1B	88.5(3)	C9B	C10B	C11B	116.9(7)
C1B^2	Fe1B	N1B^2	88.5(3)	C8B	C7B	C6B	118.5(8)
C1B	Fe1B	N1B^2	90.0(3)	C8B	C9B	C10B	118.6(8)
C1B	Fe1B	C1B^2	97.9(5)	C3	C4	N2	105.3(9)
F1B	P2	F6B	87.5(4)	C6	C7	C8	121.2(9)
F3B	P2	F1B	90.5(4)	C13B	C14B	N4B	107.3(8)
F3B	P2	F6B	86.3(5)	N5	C13	C14	107.1(8)
F4B	P2	F1B	89.5(4)	C13	C14	N4	106.4(8)
F4B	P2	F3B	93.2(5)	C3B	C4B	N2B	106.4(8)
F4B	P2	F2B	91.0(4)	N2	C5	C6	110.2(8)
F4B	P2	F5B	88.7(7)	C7	C8	C9	116.9(9)
F4B	P2	F6B	177.0(4)	N1	C6	C5	118.9(8)
F2B	P2	F1B	179.2(4)	C7	C6	N1	122.7(8)
F2B	P2	F3B	88.9(5)	C7	C6	C5	118.4(8)
F2B	P2	F5B	91.3(5)	C14B	C13B	N5B	106.0(8)
F2B	P2	F6B	92.0(5)	C4	C3	N3	106.9(9)
F5B	P2	F1B	89.3(5)	N2B	C5B	C6B	110.6(7)
F5B	P2	F3B	178.0(6)	F6A	P1A	F3A	180.0
F5B	P2	F6B	91.7(6)	F6A	P1A	F2A	90.0

C12	N4	C15	126.5(7)	F4A	P1A	F6A	90.1
C12	N4	C14	111.3(7)	F4A	P1A	F3A	89.9
C14	N4	C15	122.2(8)	F4A	P1A	F2A	90.0
C6B	N1B	Fe1B	121.8(5)	F4A	P1A	F5A	90.1
C10B	N1B	Fe1B	122.7(5)	F1A	P1A	F6A	89.9
C10B	N1B	C6B	115.5(7)	F1A	P1A	F4A	180.0(6)
C12B	N5B	C11B	122.5(7)	F1A	P1A	F3A	90.1
C12B	N5B	C13B	112.6(8)	F1A	P1A	F2A	90.0
C13B	N5B	C11B	124.8(7)	F1A	P1A	F5A	89.9
C12	N5	C11	121.5(7)	F2A	P1A	F3A	90.0
C13	N5	C12	113.3(7)	F5A	P1A	F6A	90.0
C13	N5	C11	125.2(7)	F5A	P1A	F3A	90.0
C12B	N4B	C15B	125.4(7)	F5A	P1A	F2A	180.0
C12B	N4B	C14B	111.7(8)	F4A'	P1A'	F3A'	90.7(10)
C14B	N4B	C15B	122.9(8)	F4A'	P1A'	F1A'	176.5(11)
C1B	N2B	C4B	112.1(8)	F4A'	P1A'	F2A'	92.3(10)
C1B	N2B	C5B	121.9(7)	F4A'	P1A'	F6A'	93.8(10)
C4B	N2B	C5B	126.0(7)	F3A'	P1A'	F1A'	90.6(9)
C1B	N3B	C2B	127.1(7)	F3A'	P1A'	F2A'	89.1(9)
C1B	N3B	C3B	112.5(8)	F3A'	P1A'	F6A'	174.5(12)
C3B	N3B	C2B	120.4(8)	F5A'	P1A'	F4A'	93.4(12)
C10	N1	Fe1	123.0(6)	F5A'	P1A'	F3A'	93.2(9)
C10	N1	C6	115.2(7)	F5A'	P1A'	F1A'	89.8(12)
C6	N1	Fe1	121.6(6)	F5A'	P1A'	F2A'	173.8(14)
C1	N2	C4	112.8(8)	F5A'	P1A'	F6A'	89.7(9)
C1	N2	C5	122.0(7)	F2A'	P1A'	F1A'	84.4(10)
C4	N2	C5	125.0(8)	F2A'	P1A'	F6A'	87.5(9)
N4	C12	Fe1	135.1(6)	F6A'	P1A'	F1A'	84.8(10)
N4	C12	N5	101.8(7)				

Table S6 Torsion Angles for C1.

A	B	C	D	Angle/°	A	B	C	D	Angle/°
Fe1	N1	C10	C9	-174.7(7)	C11	C10	C9	C8	-175.1(9)
Fe1	N1	C10	C11	3.3(12)	C1	N2	C4	C3	-2.9(11)
Fe1	N1	C6	C7	172.0(8)	C1	N2	C5	C6	-55.8(11)
Fe1	N1	C6	C5	-6.4(12)	C1	N3	C3	C4	-0.6(11)
Fe1B	N1B	C6B	C7B	174.4(7)	C10B	N1B	C6B	C7B	-2.5(13)
Fe1B	N1B	C6B	C5B	-2.6(11)	C10B	N1B	C6B	C5B	-179.5(8)
Fe1B	N1B	C10B	C11B	9.3(11)	C7B	C6B	C5B	N2B	132.3(9)
Fe1B	N1B	C10B	C9B	-172.0(7)	C7B	C8B	C9B	C10B	-1.1(17)
N1B	C6B	C7B	C8B	-1.5(15)	C15B	N4B	C12B	Fe1B	2.8(13)
N1B	C6B	C5B	N2B	-50.4(10)	C15B	N4B	C12B	N5B	-179.6(7)
N1B	C10B	C9B	C8B	-3.3(15)	C15B	N4B	C14B	C13B	179.8(8)
N5B	C11B	C10B	N1B	-55.4(11)	C9B	C8B	C7B	C6B	3.4(17)
N5B	C11B	C10B	C9B	125.8(9)	C4	N2	C1	Fe1	-171.2(6)
N5	C13	C14	N4	-1.0(12)	C4	N2	C1	N3	2.5(9)
N4B	C14B	C13B	N5B	-0.9(11)	C4	N2	C5	C6	118.2(9)
N3B	C3B	C4B	N2B	0.1(9)	C15	N4	C12	Fe1	-2.1(15)

N1	C10	C9	C8	3.0(16)	C15	N4	C12	N5	180.0(8)
N1	C10	C11	N5	47.9(12)	C15	N4	C14	C13	-179.5(9)
N2	C4	C3	N3	2.0(11)	C14B	N4B	C12B	Fe1B	-178.9(7)
N2	C5	C6	N1	56.0(12)	C14B	N4B	C12B	N5B	-1.4(9)
N2	C5	C6	C7	-122.4(10)	C13	N5	C12	Fe1	-178.2(7)
C12	N4	C14	C13	1.0(11)	C13	N5	C12	N4	0.0(10)
C12	N5	C11	C10	-52.3(11)	C13	N5	C11	C10	129.1(10)
C12	N5	C13	C14	0.6(12)	C2	N3	C1	Fe1	-11.3(14)
C6B	N1B	C10B	C11B	-173.8(8)	C2	N3	C1	N2	176.3(8)
C6B	N1B	C10B	C9B	5.0(13)	C2	N3	C3	C4	-178.2(8)
C12B	N5B	C11B	C10B	50.7(10)	C14	N4	C12	Fe1	177.3(7)
C12B	N5B	C13B	C14B	0.1(10)	C14	N4	C12	N5	-0.7(10)
C12B	N4B	C14B	C13B	1.5(11)	C4B	N2B	C1B	Fe1B	168.6(6)
C1B	N2B	C4B	C3B	1.2(10)	C4B	N2B	C1B	N3B	-1.9(9)
C1B	N2B	C5B	C6B	58.7(10)	C4B	N2B	C5B	C6B	-119.4(8)
C1B	N3B	C3B	C4B	-1.3(10)	C5	N2	C1	Fe1	3.5(11)
C10	N1	C6	C7	-2.8(14)	C5	N2	C1	N3	177.2(7)
C10	N1	C6	C5	178.8(8)	C5	N2	C4	C3	-177.4(8)
C10	C9	C8	C7	-3.1(16)	C8	C7	C6	N1	2.6(17)
C2B	N3B	C1B	Fe1B	12.0(14)	C8	C7	C6	C5	-179.1(11)
C2B	N3B	C1B	N2B	-179.6(8)	C6	N1	C10	C9	0.0(13)
C2B	N3B	C3B	C4B	-179.9(8)	C6	N1	C10	C11	178.1(8)
C9	C10	C11	N5	-133.9(9)	C6	C7	C8	C9	0.5(17)
C3B	N3B	C1B	Fe1B	-166.5(7)	C13B	N5B	C12B	Fe1B	178.7(6)
C3B	N3B	C1B	N2B	1.9(9)	C13B	N5B	C12B	N4B	0.8(9)
C11B	N5B	C12B	Fe1B	1.0(10)	C13B	N5B	C11B	C10B	-126.8(8)
C11B	N5B	C12B	N4B	-177.0(7)	C3	N3	C1	Fe1	171.3(7)
C11B	N5B	C13B	C14B	177.8(8)	C3	N3	C1	N2	-1.1(9)
C11B	C10B	C9B	C8B	175.5(10)	C5B	N2B	C1B	Fe1B	-9.7(10)
C11	N5	C12	Fe1	3.1(11)	C5B	N2B	C1B	N3B	179.8(7)
C11	N5	C12	N4	-178.7(8)	C5B	N2B	C4B	C3B	179.4(8)
C11	N5	C13	C14	179.3(9)	C5B	C6B	C7B	C8B	175.5(10)

Table S7 Hydrogen Atom Coordinates (Å×10^4) and Isotropic Displacement Parameters (Å^2×10^3) for C1.

Atom	*x*	*y*	*z*	U(eq)
H8B	-2150.12	10505.79	-143.94	63
H2BA	-5333.44	9925.82	-4357.5	62
H2BB	-5437.49	11292.36	-4893.89	62
H2BC	-5910.35	11151.83	-4472.85	62
H9	2345.48	6859.74	3165.93	58
H3B	-4500.91	13105.9	-4378.86	56
H11C	-4045.94	7318.05	-2015.79	45
H11D	-3689.3	6871.56	-1155.42	45
H11A	1325.61	5680.99	2589.05	51
H11B	847.6	6106.67	2979.16	51
H7B	-2656.03	12561.16	-878.73	55
H15D	-6268.37	10240.39	-1910.89	71

H15E	-6624.23	8833.31	-1748.81	71
H15F	-6642.51	9026.75	-2541.85	71
H9B	-2755.42	8285.19	-439.04	54
H4	1117	13206.14	4343.37	63
H7	2458.23	11166.24	3660.69	61
H15A	-1579.86	7901.63	674.45	67
H15B	-1322.44	8153.8	56.24	67
H15C	-1115.69	9310.18	717.76	67
H14B	-5839.45	6739.82	-1102.27	61
H13	517.33	4931.75	1282.76	58
H2A	-707.08	9093.3	3639.09	79
H2B	-1141.85	10597.15	3420.96	79
H2C	-665.79	10186.45	4275.54	79
H14	-584.85	5870.04	256.79	54
H4B	-3722.46	14146.66	-3145.14	52
H5A	1492.83	12559.82	3352.55	52
H5B	839.3	12094.86	2582.24	52
H8	3044.02	8956.83	3718.34	66
H13B	-4652.14	5919.36	-845.82	56
H3	178.33	12333.23	4658.63	65
H5BA	-4280.11	13293.74	-1812.13	44
H5BB	-3557.54	13725.23	-1820.68	44

Table S8 Atomic Occupancy for C1.

Atom	*Occupancy*	Atom	*Occupancy*	Atom	*Occupancy*
P1A	0.532(5)	F6A	0.532(5)	F4A	0.532(5)
F3A	0.532(5)	F1A	0.532(5)	F2A	0.532(5)
F5A	0.532(5)	P1A'	0.468(5)	F4A'	0.468(5)
F3A'	0.468(5)	F1A'	0.468(5)	F5A'	0.468(5)
F2A'	0.468(5)	F6A'	0.468(5)		

Complex C2

Table S9 Crystal data and structure refinement for C2.

Identification code	C2
Empirical formula	$C_{46}H_{34}F_{12}FeN_6O_2P_2$
Formula weight	1048.58
Temperature/K	110(1)
Crystal system	monoclinic
Space group	$P2_1/c$
a/Å	11.6586(10)
b/Å	26.4206(11)
c/Å	19.0403(17)
α/°	90
β/°	133.472(15)

γ/°	90
Volume/Å^3	4256.2(10)
Z	4
ρ_{calc}g/cm^3	1.636
μ/mm^{-1}	0.532
F(000)	2128.0
Crystal size/mm^3	0.323 × 0.175 × 0.141
Radiation	MoKα (λ = 0.71073)
2Θ range for data collection/°	4.266 to 54.998
Index ranges	-15 ≤ h ≤ 15, -34 ≤ k ≤ 31, -24 ≤ l ≤ 19
Reflections collected	43693
Independent reflections	9772 [R_{int} = 0.0374, R_{sigma} = 0.0306]
Data/restraints/parameters	9772/155/630
Goodness-of-fit on F^2	1.098
Final R indexes [I>=2σ (I)]	R_1 = 0.0489, wR_2 = 0.1080
Final R indexes [all data]	R_1 = 0.0602, wR_2 = 0.1147
Largest diff. peak/hole / e Å^{-3}	0.89/-0.60

Table S10 Fractional Atomic Coordinates (×10^4) and Equivalent Isotropic Displacement Parameters (Å^2×10^3) for C2. U_{eq} is defined as 1/3 of of the trace of the orthogonalised U_{IJ} tensor.

Atom	*x*	*y*	*z*	U(eq)
Fe1	3351.3(4)	3620.4(2)	5285.6(2)	11.34(8)
P1A	-1679.7(8)	3128.2(3)	5451.6(5)	16.88(14)
F4A	-968.1(19)	2581.7(6)	5560.9(12)	26.7(4)
F6A	-50.4(19)	3275.0(6)	6521.2(11)	26.9(4)
F3A	-2390(2)	3672.1(6)	5345.9(12)	28.1(4)
F1A	-1007.6(19)	3345.3(7)	5009.7(12)	29.0(4)
F5A	-3307.9(18)	2980.7(7)	4386.0(11)	26.7(4)
F2A	-2340(2)	2904.7(6)	5898.9(12)	27.7(4)
N3'	3321(2)	3376.0(8)	6264.2(14)	13.3(4)
N3	1065(2)	3769.5(8)	4436.6(14)	13.0(4)
N1	5663(2)	3476.1(8)	6187.7(14)	12.8(4)
N1'	3395(2)	3856.3(8)	4307.8(15)	14.7(4)
N2	2846(2)	2941.9(7)	4720.5(14)	12.7(4)
N2'	3818(2)	4308.1(8)	5802.5(15)	14.6(4)
C7	25(3)	3659.6(9)	3465.3(17)	14.3(5)
C2	6212(3)	3039.4(9)	6727.4(17)	13.7(5)
C5	1576(3)	2864.5(9)	3757.8(18)	15.1(5)
C8	588(3)	4074.3(9)	4749.3(18)	15.5(5)
C11'	4991(3)	3189.1(10)	7972.1(18)	18.7(5)
C3	5236(3)	2603.7(9)	6324.8(17)	14.5(5)
C7'	4541(3)	3509.2(9)	7220.1(18)	15.8(5)
C15	1307(3)	2391.7(10)	3336.4(18)	18.5(5)
C5'	4721(3)	4392.5(10)	6760.2(18)	17.7(5)
C11	-1401(3)	3942.1(10)	2794.4(18)	18.7(5)
C8'	2389(3)	2997.2(9)	6067.2(18)	15.4(5)
C16	2375(3)	2005.0(10)	3881(2)	22.1(5)
C6	382(3)	3268.3(9)	3121.9(17)	15.2(5)
C17	3657(3)	2082.5(10)	4854.0(19)	19.3(5)
C21	7731(3)	3020.5(10)	7678.3(18)	18.4(5)
C1	6704(3)	3840.0(10)	6492.9(18)	16.7(5)

C6'	5378(3)	3970.6(10)	7459.8(18)	17.4(5)
C18	5711(3)	2192.5(10)	6920.7(19)	19.1(5)
C12	-2354(3)	3873.6(11)	1790.9(19)	24.3(6)
C9'	2656(3)	2697.2(10)	6776.8(19)	19.7(5)
C4	3852(3)	2548.0(9)	5269.3(18)	15.2(5)
C23	8275(3)	3826.5(10)	7383.8(19)	20.8(5)
C14	-579(3)	3228.8(10)	2130.1(19)	20.6(5)
C2'	2475(3)	4266.6(9)	3744.6(18)	16.4(5)
C18'	999(3)	4995.0(10)	3497(2)	23.5(6)
C19	7142(3)	2194.2(10)	7898.6(19)	21.1(5)
C4'	3194(3)	4712.3(9)	5189.5(19)	17.6(5)
C20	8149(3)	2594.2(10)	8264.1(18)	21.3(5)
C12'	6416(3)	3285.8(11)	8942.3(19)	24.6(6)
C23'	3422(3)	3611.3(10)	3098.4(19)	20.3(5)
C14'	6760(3)	4046.9(11)	8416.1(19)	23.4(6)
C22	8753(3)	3435.9(10)	8002.2(19)	21.0(5)
C1'	3906(3)	3558.9(9)	4005.0(18)	16.3(5)
C22'	2296(3)	3963.7(11)	2465.5(19)	22.5(6)
C13'	7298(3)	3697.5(11)	9147.7(19)	27.6(6)
C10'	3994(3)	2777.2(10)	7723.2(19)	20.8(5)
C20'	600(3)	4674.7(11)	2158(2)	25.6(6)
C16'	4402(3)	5292.4(10)	6508(2)	24.2(6)
C3'	2182(3)	4645.9(10)	4135.2(19)	18.4(5)
C15'	4995(3)	4883.2(10)	7123(2)	23.1(6)
C10	-1810(3)	4285.0(10)	3158.9(19)	21.6(5)
C13	-1920(3)	3536.1(11)	1470.8(19)	25.3(6)
C9	-858(3)	4332.4(10)	4134.2(19)	20.4(5)
C21'	1783(3)	4305.3(10)	2776.3(19)	21.0(5)
C19'	169(3)	4994.0(11)	2504(2)	26.3(6)
C17'	3518(3)	5206.9(10)	5540(2)	21.9(5)
P1B	7335.3(10)	5691.2(3)	9612.2(5)	28.13(17)
F1B	6838(3)	5127.5(8)	9610(2)	61.0(7)
F5B	5661(3)	5780.8(10)	8552.2(17)	74.3(9)
F3B	7821(3)	6253.5(8)	9619.3(15)	51.0(6)
F6B	8981(3)	5604.7(9)	10664.1(14)	70.8(8)
F2B	6615(4)	5887.7(9)	10042(2)	79.4(9)
F4B	7973(3)	5478.2(8)	9155.9(16)	48.3(5)
O2W	1684(4)	4222.5(11)	6844(2)	53.9(7)
O1W	3985(6)	4547(3)	8809(4)	147(2)

Table S11 Anisotropic Displacement Parameters (Å^2×10^3) for C2. The Anisotropic displacement factor exponent takes the form: $-2\pi^2[h^2a^{*2}U_{11}+2hka^*b^*U_{12}+\ldots]$.

Atom	U_{11}	U_{22}	U_{33}	U_{23}	U_{13}	U_{12}
Fe1	10.34(16)	11.22(16)	11.15(16)	-0.44(12)	6.89(14)	-0.71(12)
P1A	15.6(3)	18.5(3)	16.8(3)	2.1(2)	11.3(3)	0.9(2)
F4A	20.8(8)	23.8(8)	33.2(9)	-2.0(7)	17.7(7)	3.9(7)
F6A	23.8(8)	29.1(9)	17.3(8)	-0.4(6)	10.1(7)	-0.9(7)
F3A	30.6(9)	18.2(8)	33.1(9)	5.0(7)	21.0(8)	6.2(7)
F1A	23.6(8)	42.6(10)	24.9(8)	4.2(7)	18.2(7)	-4.8(7)
F5A	15.5(7)	35.7(9)	20.8(8)	-2.6(7)	9.4(7)	-1.7(7)
F2A	34.2(9)	27.6(9)	36.0(9)	6.0(7)	29.8(8)	2.3(7)
N3'	13.2(9)	14.6(10)	14.4(9)	0.2(8)	10.4(8)	2.2(8)

N3	12.9(9)	13.3(10)	13.7(9)	0.8(7)	9.5(8)	-0.8(8)
N1	11.9(9)	14.5(10)	12.6(9)	-1.4(7)	8.6(8)	-0.4(8)
N1'	12.0(9)	13.6(10)	16.5(10)	0.5(8)	9.1(8)	-1.5(8)
N2	13.0(9)	12.6(10)	14.4(9)	-0.8(7)	10.2(8)	-2.2(8)
N2'	11.7(9)	12.9(10)	18.1(10)	-1.5(8)	9.8(9)	-1.7(8)
C7	12.1(11)	18.6(12)	13.7(11)	0.5(9)	9.4(10)	-2.9(9)
C2	14.4(11)	15.4(11)	14.8(11)	-0.5(9)	11.3(10)	2.4(9)
C5	15.1(11)	15.7(12)	15.9(11)	-2.2(9)	11.2(10)	-3.9(9)
C8	13.2(11)	15.7(12)	16.8(11)	0.4(9)	9.9(10)	-0.5(9)
C11'	21.6(13)	20.7(13)	16.1(12)	0.6(10)	13.9(11)	5.5(10)
C3	15.8(11)	15.8(12)	16.2(11)	0.4(9)	12.6(10)	2.3(9)
C7'	15.2(11)	16.9(12)	15.3(11)	-0.9(9)	10.4(10)	3.1(9)
C15	18.4(12)	20.8(13)	16.8(12)	-5.5(10)	12.3(11)	-5.0(10)
C5'	14.9(11)	17.6(12)	19.5(12)	-3.5(10)	11.5(10)	-1.4(10)
C11	12.8(11)	21.4(13)	17.8(12)	3.3(10)	8.9(10)	0.0(10)
C8'	12.9(11)	17.7(12)	16.2(11)	2.7(9)	10.2(10)	2.2(9)
C16	24.9(14)	15.8(12)	25.9(14)	-7.3(10)	17.5(12)	-3.7(10)
C6	12.1(11)	18.7(12)	14.5(11)	-1.1(9)	9.0(10)	-4.4(9)
C17	21.6(13)	15.0(12)	23.3(13)	-1.6(10)	16.2(11)	-0.4(10)
C21	14.6(12)	22.0(13)	16.2(12)	-0.8(10)	9.6(10)	4.1(10)
C1	15.2(12)	16.9(12)	17.8(12)	-2.6(9)	11.3(10)	-1.5(9)
C6'	17.2(12)	18.6(12)	15.6(11)	-3.0(9)	11.0(10)	0.5(10)
C18	22.9(13)	15.3(12)	23.0(13)	1.9(10)	17.3(11)	3.4(10)
C12	15.6(12)	28.8(15)	17.2(12)	5.5(11)	6.9(11)	1.5(11)
C9'	18.8(12)	20.2(13)	25.2(13)	4.8(10)	17.1(11)	3.5(10)
C4	15.7(11)	14.3(12)	17.4(11)	0.5(9)	12.0(10)	-0.3(9)
C23	14.3(12)	22.0(13)	21.8(13)	-5.0(10)	10.8(11)	-4.1(10)
C14	18.8(12)	24.4(13)	16.7(12)	-2.9(10)	11.5(11)	-4.0(10)
C2'	13.3(11)	16.1(12)	18.4(12)	4.0(9)	10.3(10)	-1.7(9)
C18'	20.9(13)	18.2(13)	30.5(14)	7.5(11)	17.3(12)	3.2(10)
C19	26.3(14)	22.8(13)	21.4(13)	7.6(10)	19.2(12)	11.1(11)
C4'	14.5(11)	14.8(12)	24.1(13)	1.2(10)	13.5(11)	0.2(9)
C20	19.6(13)	26.7(14)	14.6(11)	2.5(10)	10.6(11)	8.8(11)
C12'	27.8(14)	27.3(15)	13.6(12)	1.0(10)	12.2(12)	6.4(12)
C23'	19.5(12)	25.3(14)	20.4(12)	-0.7(10)	15.3(11)	-3.1(11)
C14'	22.5(13)	22.8(14)	18.5(12)	-7.9(10)	11.7(11)	-2.2(11)
C22	12.2(11)	26.5(14)	16.0(12)	-3.7(10)	6.5(10)	0.9(10)
C1'	14.0(11)	16.9(12)	17.2(11)	1.0(9)	10.4(10)	-2.2(9)
C22'	21.8(13)	29.6(14)	17.7(12)	2.0(11)	14.2(11)	-4.8(11)
C13'	24.2(14)	31.4(16)	13.4(12)	-4.4(11)	7.7(11)	2.4(12)
C10'	24.4(13)	23.6(13)	22.1(13)	6.9(10)	18.9(12)	7.1(11)
C20'	20.6(13)	27.6(15)	20.5(13)	9.8(11)	11.1(11)	1.4(11)
C16'	21.7(13)	14.9(12)	36.4(15)	-8.7(11)	20.2(13)	-5.1(10)
C3'	16.3(12)	15.1(12)	24.1(13)	3.8(10)	14.0(11)	-0.7(10)
C15'	20.8(13)	20.3(13)	23.9(13)	-7.3(11)	13.7(12)	-3.2(11)
C10	13.6(12)	25.2(14)	21.7(13)	5.3(10)	10.6(11)	4.6(10)
C13	20.7(13)	34.6(16)	11.8(11)	0.7(11)	7.8(11)	-2.0(12)
C9	18.2(12)	21.8(13)	24.6(13)	2.1(10)	16.0(11)	4.2(10)
C21'	17.8(12)	21.6(13)	20.0(12)	5.8(10)	11.7(11)	-1.7(10)
C19'	19.2(13)	23.3(14)	29.2(14)	12.6(11)	13.8(12)	3.7(11)
C17'	21.2(13)	14.3(12)	34.6(15)	1.1(11)	20.9(12)	0.5(10)

P1B	31.9(4)	25.5(4)	19.8(3)	-5.2(3)	15.0(3)	-3.8(3)
F1B	91.2(19)	30.6(11)	95.2(19)	-22.1(12)	77.1(17)	-22.5(12)
F5B	33.7(12)	74.7(18)	46.3(14)	-18.1(12)	1.4(11)	10.4(12)
F3B	58.9(14)	31.4(11)	38.7(11)	5.7(9)	24.4(11)	-7.7(10)
F6B	73.7(17)	45.7(13)	18.3(10)	7.0(9)	3.0(10)	-6.2(12)
F2B	142(3)	46.1(14)	119(2)	-19.2(15)	116(2)	-7.2(16)
F4B	56.2(13)	54.8(13)	50.5(12)	6.7(10)	43.1(12)	8.4(11)
O2W	60.8(18)	52.4(17)	52.3(16)	-4.4(13)	40.4(15)	-1.6(14)
O1W	99(4)	250(7)	89(3)	-44(4)	65(3)	-62(4)

Table S12 Bond Lengths for C2.

Atom	Atom	Length/Å	Atom	Atom	Length/Å
Fe1	N3'	1.995(2)	C5'	C15'	1.399(4)
Fe1	N3	1.986(2)	C11	C12	1.413(4)
Fe1	N1	1.997(2)	C11	C10	1.409(4)
Fe1	N1'	1.997(2)	C8'	C9'	1.404(3)
Fe1	N2	1.961(2)	C16	C17	1.378(4)
Fe1	N2'	1.957(2)	C6	C14	1.386(3)
P1A	F4A	1.6057(17)	C17	C4	1.394(3)
P1A	F6A	1.6072(17)	C21	C20	1.415(4)
P1A	F3A	1.6005(17)	C21	C22	1.412(4)
P1A	F1A	1.5994(17)	C1	C23	1.400(4)
P1A	F5A	1.6038(17)	C6'	C14'	1.384(4)
P1A	F2A	1.6024(17)	C18	C19	1.406(4)
N3'	C7'	1.377(3)	C12	C13	1.359(4)
N3'	C8'	1.328(3)	C9'	C10'	1.363(4)
N3	C7	1.374(3)	C23	C22	1.363(4)
N3	C8	1.330(3)	C14	C13	1.407(4)
N1	C2	1.376(3)	C2'	C3'	1.422(4)
N1	C1	1.328(3)	C2'	C21'	1.419(4)
N1'	C2'	1.376(3)	C18'	C3'	1.387(4)
N1'	C1'	1.333(3)	C18'	C19'	1.412(4)
N2	C5	1.364(3)	C19	C20	1.362(4)
N2	C4	1.365(3)	C4'	C3'	1.481(4)
N2'	C5'	1.357(3)	C4'	C17'	1.396(4)
N2'	C4'	1.365(3)	C12'	C13'	1.356(4)
C7	C11	1.425(3)	C23'	C1'	1.406(3)
C7	C6	1.430(3)	C23'	C22'	1.366(4)
C2	C3	1.417(3)	C14'	C13'	1.409(4)
C2	C21	1.416(3)	C22'	C21'	1.417(4)
C5	C15	1.399(3)	C20'	C21'	1.417(4)
C5	C6	1.491(3)	C20'	C19'	1.363(4)
C8	C9	1.401(3)	C16'	C15'	1.379(4)
C11'	C7'	1.417(4)	C16'	C17'	1.378(4)
C11'	C12'	1.419(4)	C10	C9	1.364(4)
C11'	C10'	1.415(4)	P1B	F1B	1.597(2)
C3	C18	1.382(3)	P1B	F5B	1.587(2)
C3	C4	1.484(3)	P1B	F3B	1.586(2)
C7'	C6'	1.426(4)	P1B	F6B	1.568(2)
C15	C16	1.376(4)	P1B	F2B	1.608(2)
C5'	C6'	1.483(4)	P1B	F4B	1.585(2)

Table S13 Bond Angles for MD180.

Atom	Atom	Atom	Angle/°	Atom	Atom	Atom	Angle/°
N3'	Fe1	N1	90.88(8)	C12	C11	C7	119.5(2)
N3'	Fe1	N1'	179.22(9)	C10	C11	C7	118.5(2)
N3	Fe1	N3'	87.04(8)	C10	C11	C12	122.0(2)
N3	Fe1	N1	177.60(8)	N3'	C8'	C9'	123.9(2)
N3	Fe1	N1'	93.45(8)	C15	C16	C17	119.0(2)
N1'	Fe1	N1	88.64(8)	C7	C6	C5	123.3(2)
N2	Fe1	N3'	91.44(8)	C14	C6	C7	117.4(2)
N2	Fe1	N3	90.38(8)	C14	C6	C5	118.7(2)
N2	Fe1	N1	90.88(8)	C16	C17	C4	119.8(2)
N2	Fe1	N1'	87.95(8)	C20	C21	C2	119.0(2)
N2'	Fe1	N3'	90.49(9)	C22	C21	C2	117.9(2)
N2'	Fe1	N3	88.77(8)	C22	C21	C20	123.0(2)
N2'	Fe1	N1	90.05(8)	N1	C1	C23	123.5(2)
N2'	Fe1	N1'	90.13(9)	C7'	C6'	C5'	122.5(2)
N2'	Fe1	N2	177.85(9)	C14'	C6'	C7'	117.8(2)
F4A	P1A	F6A	89.88(9)	C14'	C6'	C5'	119.1(2)
F3A	P1A	F4A	179.74(10)	C3	C18	C19	121.7(2)
F3A	P1A	F6A	90.04(9)	C13	C12	C11	120.1(2)
F3A	P1A	F5A	89.97(9)	C10'	C9'	C8'	118.6(2)
F3A	P1A	F2A	90.10(9)	N2	C4	C3	121.5(2)
F1A	P1A	F4A	89.67(10)	N2	C4	C17	121.3(2)
F1A	P1A	F6A	90.02(9)	C17	C4	C3	117.2(2)
F1A	P1A	F3A	90.57(10)	C22	C23	C1	118.9(2)
F1A	P1A	F5A	90.20(9)	C6	C14	C13	122.2(3)
F1A	P1A	F2A	179.30(10)	N1'	C2'	C3'	119.1(2)
F5A	P1A	F4A	90.10(9)	N1'	C2'	C21'	120.7(2)
F5A	P1A	F6A	179.78(11)	C21'	C2'	C3'	120.1(2)
F2A	P1A	F4A	89.66(9)	C3'	C18'	C19'	121.6(3)
F2A	P1A	F6A	89.80(9)	C20	C19	C18	120.0(2)
F2A	P1A	F5A	89.98(9)	N2'	C4'	C3'	121.7(2)
C7'	N3'	Fe1	119.06(16)	N2'	C4'	C17'	120.9(2)
C8'	N3'	Fe1	121.14(17)	C17'	C4'	C3'	117.4(2)
C8'	N3'	C7'	117.8(2)	C19	C20	C21	120.4(2)
C7	N3	Fe1	120.09(16)	C13'	C12'	C11'	119.7(3)
C8	N3	Fe1	119.76(16)	C22'	C23'	C1'	118.7(2)
C8	N3	C7	118.4(2)	C6'	C14'	C13'	121.9(3)
C2	N1	Fe1	117.51(16)	C23	C22	C21	119.4(2)
C1	N1	Fe1	121.61(17)	N1'	C1'	C23'	123.5(2)
C1	N1	C2	117.7(2)	C23'	C22'	C21'	119.4(2)
C2'	N1'	Fe1	117.76(17)	C12'	C13'	C14'	120.5(3)
C1'	N1'	Fe1	121.66(17)	C9'	C10'	C11'	119.4(2)
C1'	N1'	C2'	117.9(2)	C19'	C20'	C21'	119.8(3)
C5	N2	Fe1	120.85(16)	C17'	C16'	C15'	118.9(2)
C5	N2	C4	118.8(2)	C2'	C3'	C4'	122.6(2)
C4	N2	Fe1	120.09(16)	C18'	C3'	C2'	117.5(2)
C5'	N2'	Fe1	121.23(17)	C18'	C3'	C4'	119.6(2)
C5'	N2'	C4'	119.0(2)	C16'	C15'	C5'	119.8(3)
C4'	N2'	Fe1	119.75(17)	C9	C10	C11	119.7(2)
N3	C7	C11	120.1(2)	C12	C13	C14	120.4(2)
N3	C7	C6	120.2(2)	C10	C9	C8	118.5(2)
C11	C7	C6	119.7(2)	C22'	C21'	C2'	118.1(2)
N1	C2	C3	119.3(2)	C20'	C21'	C2'	119.2(3)

N1	C2	C21	121.0(2)	C20'	C21'	C22'	122.7(3)
C21	C2	C3	119.7(2)	C20'	C19'	C18'	120.5(3)
N2	C5	C15	120.8(2)	C16'	C17'	C4'	120.0(3)
N2	C5	C6	121.9(2)	F1B	P1B	F2B	89.28(13)
C15	C5	C6	117.2(2)	F5B	P1B	F1B	90.52(16)
N3	C8	C9	123.7(2)	F5B	P1B	F2B	89.02(17)
C7'	C11'	C12'	119.7(3)	F3B	P1B	F1B	179.34(14)
C10'	C11'	C7'	118.2(2)	F3B	P1B	F5B	89.46(14)
C10'	C11'	C12'	122.0(2)	F3B	P1B	F2B	90.06(14)
C2	C3	C4	122.2(2)	F6B	P1B	F1B	89.45(14)
C18	C3	C2	118.4(2)	F6B	P1B	F5B	179.35(18)
C18	C3	C4	118.8(2)	F6B	P1B	F3B	90.56(12)
N3'	C7'	C11'	120.7(2)	F6B	P1B	F2B	90.33(18)
N3'	C7'	C6'	119.9(2)	F6B	P1B	F4B	91.44(15)
C11'	C7'	C6'	119.4(2)	F4B	P1B	F1B	88.45(12)
C16	C15	C5	120.1(2)	F4B	P1B	F5B	89.21(14)
N2'	C5'	C6'	121.8(2)	F4B	P1B	F3B	92.20(13)
N2'	C5'	C15'	121.2(2)	F4B	P1B	F2B	177.11(15)
C15'	C5'	C6'	116.8(2)				

Table S14 Torsion Angles for C2.

A	B	C	D	Angle/°	A	B	C	D	Angle/°
Fe1	N3'	C7'	C11'	-150.97(19)	C7'	C11'	C10'	C9'	0.8(4)
Fe1	N3'	C7'	C6'	28.2(3)	C7'	C6'	C14'	C13'	-3.3(4)
Fe1	N3'	C8'	C9'	157.9(2)	C15	C5	C6	C7	148.9(2)
Fe1	N3	C7	C11	-152.97(18)	C15	C5	C6	C14	-22.4(3)
Fe1	N3	C7	C6	25.8(3)	C15	C16	C17	C4	-0.1(4)
Fe1	N3	C8	C9	158.3(2)	C5'	N2'	C4'	C3'	-179.2(2)
Fe1	N1	C2	C3	33.8(3)	C5'	N2'	C4'	C17'	2.3(4)
Fe1	N1	C2	C21	-145.83(19)	C5'	C6'	C14'	C13'	168.6(3)
Fe1	N1	C1	C23	151.6(2)	C11	C7	C6	C5	-162.5(2)
Fe1	N1'	C2'	C3'	30.9(3)	C11	C7	C6	C14	8.9(3)
Fe1	N1'	C2'	C21'	-147.23(19)	C11	C12	C13	C14	4.2(4)
Fe1	N1'	C1'	C23'	154.3(2)	C11	C10	C9	C8	6.4(4)
Fe1	N2	C5	C15	173.50(18)	C8'	N3'	C7'	C11'	13.1(3)
Fe1	N2	C5	C6	-9.3(3)	C8'	N3'	C7'	C6'	-167.7(2)
Fe1	N2	C4	C3	7.5(3)	C8'	C9'	C10'	C11'	6.2(4)
Fe1	N2	C4	C17	-169.85(19)	C16	C17	C4	N2	-3.9(4)
Fe1	N2'	C5'	C6'	-0.9(3)	C16	C17	C4	C3	178.7(2)
Fe1	N2'	C5'	C15'	-177.02(19)	C6	C7	C11	C12	-7.4(4)
Fe1	N2'	C4'	C3'	-1.0(3)	C6	C7	C11	C10	172.9(2)
Fe1	N2'	C4'	C17'	-179.50(19)	C6	C5	C15	C16	179.1(2)
N3'	C7'	C6'	C5'	19.5(4)	C6	C14	C13	C12	-2.5(4)
N3'	C7'	C6'	C14'	-168.8(2)	C21	C2	C3	C18	8.6(3)
N3'	C8'	C9'	C10'	-3.9(4)	C21	C2	C3	C4	-162.8(2)
N3	C7	C11	C12	171.4(2)	C1	N1	C2	C3	-166.0(2)
N3	C7	C11	C10	-8.3(4)	C1	N1	C2	C21	14.3(3)
N3	C7	C6	C5	18.7(3)	C1	C23	C22	C21	9.1(4)
N3	C7	C6	C14	-169.9(2)	C6'	C5'	C15'	C16'	-179.4(2)
N3	C8	C9	C10	-2.5(4)	C6'	C14'	C13'	C12'	-4.0(4)
N1	C2	C3	C18	-171.0(2)	C18	C3	C4	N2	146.6(2)

N1	C2	C3	C4	17.5(3)	C18	C3	C4	C17	-35.9(3)
N1	C2	C21	C20	169.7(2)	C18	C19	C20	C21	3.1(4)
N1	C2	C21	C22	-9.3(4)	C12	C11	C10	C9	179.2(3)
N1	C1	C23	C22	-4.0(4)	C4	N2	C5	C15	-0.3(3)
N1'	C2'	C3'	C18'	-166.8(2)	C4	N2	C5	C6	176.9(2)
N1'	C2'	C3'	C4'	20.0(4)	C4	C3	C18	C19	170.2(2)
N1'	C2'	C21'	C22'	-11.4(4)	C2'	N1'	C1'	C23'	-6.8(4)
N1'	C2'	C21'	C20'	167.5(2)	C4'	N2'	C5'	C6'	177.3(2)
N2	C5	C15	C16	-3.6(4)	C4'	N2'	C5'	C15'	1.2(4)
N2	C5	C6	C7	-28.4(4)	C20	C21	C22	C23	178.3(3)
N2	C5	C6	C14	160.3(2)	C12'	C11'	C7'	N3'	168.8(2)
N2'	C5'	C6'	C7'	-35.7(4)	C12'	C11'	C7'	C6'	-10.4(4)
N2'	C5'	C6'	C14'	152.7(2)	C12'	C11'	C10'	C9'	-178.7(2)
N2'	C5'	C15'	C16'	-3.0(4)	C23'	C22'	C21'	C2'	0.1(4)
N2'	C4'	C3'	C2'	-37.9(4)	C23'	C22'	C21'	C20'	-178.8(3)
N2'	C4'	C3'	C18'	149.0(2)	C22	C21	C20	C19	-177.0(2)
N2'	C4'	C17'	C16'	-3.9(4)	C1'	N1'	C2'	C3'	-167.2(2)
C7	N3	C8	C9	-6.9(4)	C1'	N1'	C2'	C21'	14.6(3)
C7	C11	C12	C13	0.8(4)	C1'	C23'	C22'	C21'	7.4(4)
C7	C11	C10	C9	-1.1(4)	C22'	C23'	C1'	N1'	-4.3(4)
C7	C6	C14	C13	-4.1(4)	C10'	C11'	C7'	N3'	-10.8(4)
C2	N1	C1	C23	-7.7(4)	C10'	C11'	C7'	C6'	170.0(2)
C2	C3	C18	C19	-1.5(4)	C10'	C11'	C12'	C13'	-177.3(3)
C2	C3	C4	N2	-42.0(3)	C3'	C2'	C21'	C22'	170.5(2)
C2	C3	C4	C17	135.5(2)	C3'	C2'	C21'	C20'	-10.6(4)
C2	C21	C20	C19	4.0(4)	C3'	C18'	C19'	C20'	-5.6(4)
C2	C21	C22	C23	-2.7(4)	C3'	C4'	C17'	C16'	177.6(2)
C5	N2	C4	C3	-178.6(2)	C15'	C5'	C6'	C7'	140.6(3)
C5	N2	C4	C17	4.0(3)	C15'	C5'	C6'	C14'	-31.0(4)
C5	C15	C16	C17	3.7(4)	C15'	C16'	C17'	C4'	1.9(4)
C5	C6	C14	C13	167.7(2)	C10	C11	C12	C13	-179.5(3)
C8	N3	C7	C11	12.1(3)	C21'	C2'	C3'	C18'	11.4(4)
C8	N3	C7	C6	-169.1(2)	C21'	C2'	C3'	C4'	-161.9(2)
C11'	C7'	C6'	C5'	-161.3(2)	C21'	C20'	C19'	C18'	6.5(4)
C11'	C7'	C6'	C14'	10.4(4)	C19'	C18'	C3'	C2'	-3.4(4)
C11'	C12'	C13'	C14'	4.1(4)	C19'	C18'	C3'	C4'	170.1(2)
C3	C2	C21	C20	-9.9(4)	C19'	C20'	C21'	C2'	1.5(4)
C3	C2	C21	C22	171.1(2)	C19'	C20'	C21'	C22'	-179.6(3)
C3	C18	C19	C20	-4.4(4)	C17'	C4'	C3'	C2'	140.7(3)
C7'	N3'	C8'	C9'	-5.8(4)	C17'	C4'	C3'	C18'	-32.5(4)
C7'	C11'	C12'	C13'	3.1(4)	C17'	C16'	C15'	C5'	1.4(4)

Table S15 Hydrogen Atom Coordinates (Å×10^4) and Isotropic Displacement Parameters (Å^2×10^3) for C2.

Atom	*x*	*y*	*z*	U(eq)
H8	1265.33	4119.47	5427.65	19
H15	385.73	2337.55	2673.22	22
H8'	1488.48	2924.76	5407.28	19
H16	2230.96	1689.38	3589.73	27

H17	4404.86	1819.4	5240.7	23
H1	6363.76	4124.56	6084.66	20
H18	5053.72	1901.31	6662.93	23
H12	-3299.54	4063.79	1339.49	29
H9'	1918.6	2443.82	6600.78	24
H23	8996.13	4084.96	7554.66	25
H14	-323.35	2986.27	1887.49	25
H18'	741.32	5240.79	3734.02	28
H19	7407.45	1916.4	8304.35	25
H20	9138.93	2586.99	8916.57	26
H12'	6750.86	3062.99	9445.62	30
H23'	3870.23	3405.76	2929.4	24
H14'	7363.47	4343.62	8584.05	28
H22	9764.46	3443.22	8644.9	25
H1'	4635.47	3297.67	4423.83	20
H22'	1859.33	3979.53	1822.46	27
H13'	8285.02	3750.58	9789.55	33
H10'	4254.51	2558.39	8211.66	25
H20'	109.51	4699.84	1503.35	31
H16'	4600.08	5627.67	6749.02	29
H15'	5586.6	4934.48	7790.28	28
H10	-2744.97	4482.34	2727.13	26
H13	-2526.56	3507.4	797.33	30
H9	-1169.18	4535.96	4390.7	24
H19'	-697.9	5217.73	2074.26	32
H17'	3128.48	5484.68	5110.6	26
H2WA	2310.18	3997.06	6956.64	1900(500)
H2WB	1277.2	4115.97	7056	1900(500)
H1WA	3600.7	4250.44	8670.84	2000(600)
H1WB	4723.36	4569.58	9424.57	2000(600)

Transient spectroscopy details

Table S16. Fit parameters for C1 determined by multi-exponential fits at single wavelengths. Amplitudes are in ΔA units multiplied by 1000.

Wavelength (nm)	τ_1 (ps)	τ_2 (ps)	τ_3 (ps)	A_1	A_2	A_3
336	0.3 (f)	1.3	4.6	-1.8	3.7	1.0
370	0.3 (f)	1.6	5.0	-0.4	2.7	0.2
450	-	1.3	7.2	-	-6.8	-1.8
510	0.4	1.7	7.5	-5.9	-9.2	-6.0
535	0.6	3.5	18.4	-5.8	-2.6	0.9
576	0.4	0.7	8.5	-10.0	8.0	1.0
600	0.4	1.3	24.3	-1.9	3.3	0.1
630	0.4	1.0	7.1	-2.0	3.0	0.04

Quality of global fit approach C1

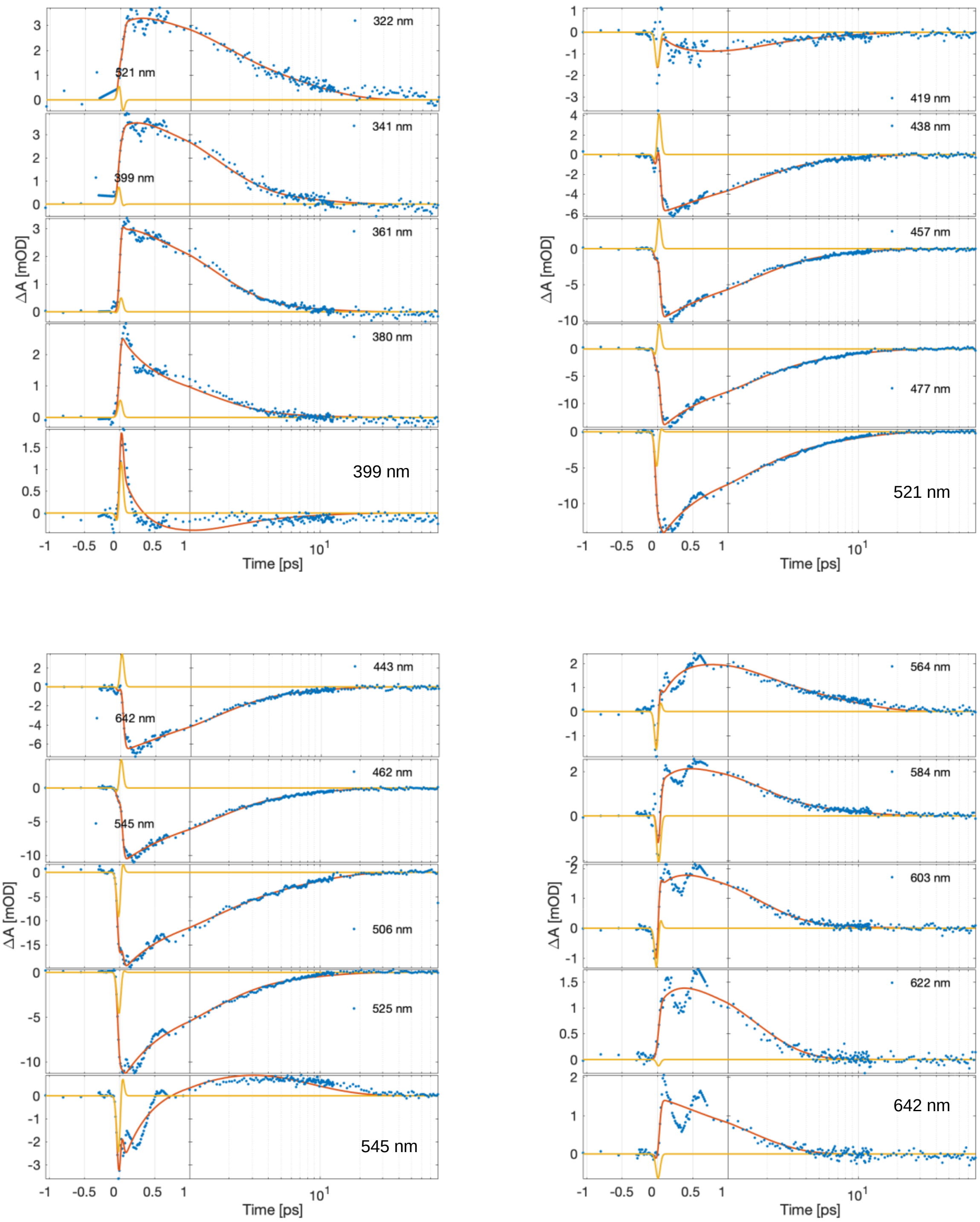

Figure S7 : Comparison of the exp. kinetic traces (blue dots), and the 3-exponential model used for global and target analysis (red line). The OPTIMUS software fits a residual non-corrected coherent artifact at time zero (yellow line). Apart from the initial 0.8 ps, dominated by the pronounced vibrational coherences, the fits are as good as single wavelength fits with wavelength-dependent lifetimes. Global lifetimes are 0.3±0.1, 1.3±0.2 and 7.3±0.5 ps. The corresponding DADS and SADS are shown in fig. 6 in the main text.

Analysis of vibrational coherences in C1

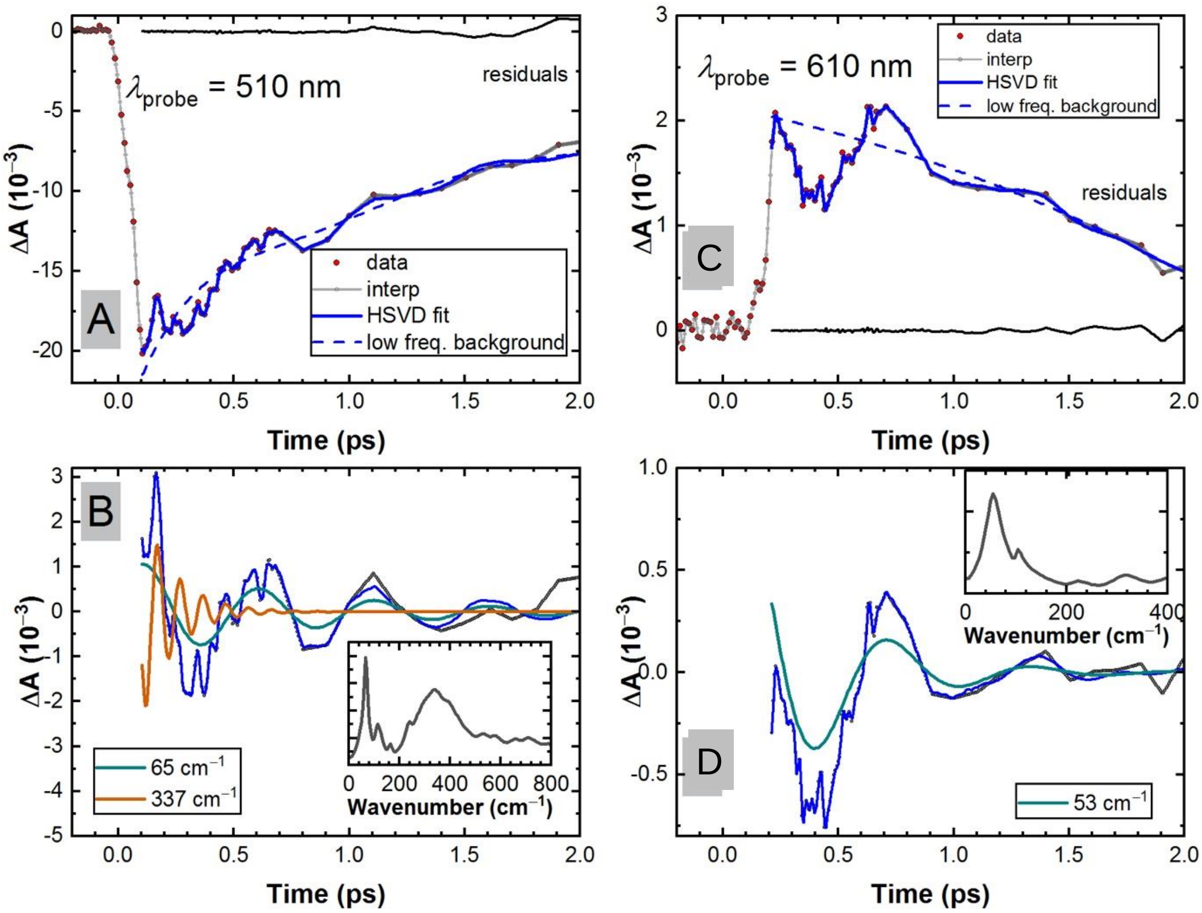

Figure S8 : Analysis of vibrational coherences in maximum of GSB (510 nm, panel A&D) and on long-wavelength wing of ESA (610 nm, C&D). Raw data (red dots) are interpolated on a finer time grid (grey line). Oscillatory features with a 0.48 ps period and a faster sub-100 fs period are discernible above the noise level (defined at t< 0). A & C: A LPSVD analysis [1] implemented in a home-made software fits the oscillations and the incoherent 3-exp. decay (dashed blue line). The best fit to the data (blue solid line), and the fit residuals (black) are shown. B&D : Focus on the oscillatory part of the LPSVD fit, and its Fourier spectrum (inset) as obtained by the LP-SVD analysis. The low frequency oscillations (53-65 cm-1) are highlighted (green) as well as the dominant high-frequency mode (≈340 cm^{-1}). The latter is consistent with Fe-N stretch vibrations, most probably due to pump-induced ground state Raman coherences. The ≈60 cm^{-1} mode is a reaction-induced excited state mode, as it appears most strongly in the ESA region. Its observation in the regions of $\Delta A < 0$, e.g. 510 nm, is due to ESA partially overlapping with the GSB.

Global fit results for C2

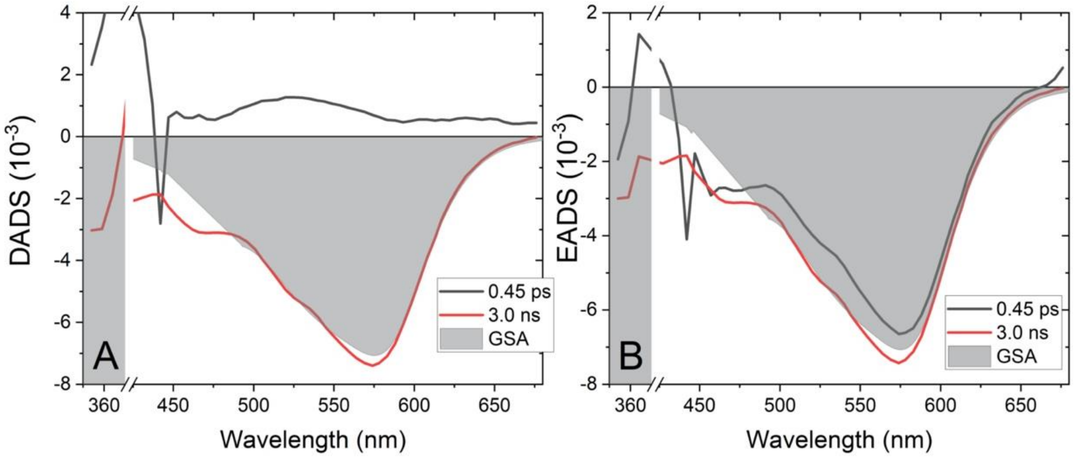

Figure S9: Results of global (A) and target analysis (B) of the TAS data of **C2**. Scaled ground state absorption spectrum in grey. The DADS of the 0.45 ps component (A) indicates a broad excited state absorption, which decays into the equilibrated quintet difference spectrum dominated by GSB in whole wavelength region.

Additional computational data

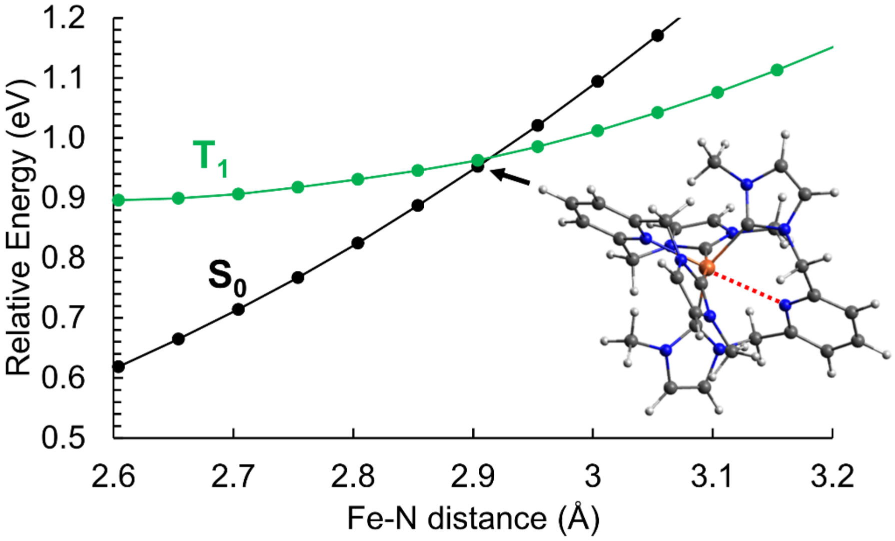

Figure S10: Scan of one Fe-N bond distance of **C1** relaxing the T_1 state. The triplet potential energy surface shows a small energy barrier (<0.05 eV) in the path towards the singlet-triplet crossing between the T_1 (^{3}MC) and the ground state.

References

1. Barkhuijsen, H.; de Beer, R.; Bovée, W.M.M.J.; van Ormondt, D. Retrieval of frequencies, amplitudes, damping factors, and phases from time-domain signals using a linear least-squares procedure. *J. Magn. Reson. 1969* **1985**, *61*, 465–481, doi:10.1016/0022-2364(85)90187-8.
